# Large-scale variations in the dynamics of Amazon forest canopy gaps from airborne lidar data and opportunities for tree mortality estimates

**DOI:** 10.1038/s41598-020-80809-w

**Published:** 2021-01-14

**Authors:** Ricardo Dalagnol, Fabien H. Wagner, Lênio S. Galvão, Annia S. Streher, Oliver L. Phillips, Emanuel Gloor, Thomas A. M. Pugh, Jean P. H. B. Ometto, Luiz E. O. C. Aragão

**Affiliations:** 1 Earth Observation and Geoinformatics Division, National Institute for Space Research-INPE, São José dos Campos, SP 12227-010 Brazil; 2 GeoProcessing Division, Foundation for Science, Technology and Space Applications-FUNCATE, São José dos Campos, SP 12210-131 Brazil; 3 School of Geography, University of Leeds, Leeds, LS2 9JT UK; 4 School of Geography, Earth and Environmental Sciences, University of Birmingham, Birmingham, B15 2TT UK; 5 Birmingham Institute of Forest Research, University of Birmingham, Birmingham, B15 2TT UK; 6 Earth System Sciences Center, National Institute for Space Research-INPE, São José dos Campos, SP 12227-010 Brazil; 7 Geography, College of Life and Environmental Sciences, University of Exeter, Exeter, EX4 4RJ UK

**Keywords:** Ecology, Ecological modelling, Forest ecology, Forestry, Macroecology, Population dynamics, Tropical ecology

## Abstract

We report large-scale estimates of Amazonian gap dynamics using a novel approach with large datasets of airborne light detection and ranging (lidar), including five multi-temporal and 610 single-date lidar datasets. Specifically, we (1) compared the fixed height and relative height methods for gap delineation and established a relationship between static and dynamic gaps (newly created gaps); (2) explored potential environmental/climate drivers explaining gap occurrence using generalized linear models; and (3) cross-related our findings to mortality estimates from 181 field plots. Our findings suggest that static gaps are significantly correlated to dynamic gaps and can inform about structural changes in the forest canopy. Moreover, the relative height outperformed the fixed height method for gap delineation. Well-defined and consistent spatial patterns of dynamic gaps were found over the Amazon, while also revealing the dynamics of areas never sampled in the field. The predominant pattern indicates 20–35% higher gap dynamics at the west and southeast than at the central-east and north. These estimates were notably consistent with field mortality patterns, but they showed 60% lower magnitude likely due to the predominant detection of the broken/uprooted mode of death. While topographic predictors did not explain gap occurrence, the water deficit, soil fertility, forest flooding and degradation were key drivers of gap variability at the regional scale. These findings highlight the importance of lidar in providing opportunities for large-scale gap dynamics and tree mortality monitoring over the Amazon.

## Introduction

There is increasing evidence showing that tree mortality is a key factor for understanding carbon storage and sequestration in the Amazon forests^1, 2^. Specifically, the majority of the mortality events are related to very frequent small- to intermediate- scale disturbances (< 5 ha) rather than episodic large-scale events (> 5 ha)^3^. However, the uncertainties associated with tree mortality drivers and mechanisms, especially at smaller scales (< 1 ha), constrain our ability to accurately measure tropical forest carbon budget and assess climate change effects^4^.

Tree mortality in the Amazon has been apparently increasing since the past decade likely due to greater climate variability and feedbacks of faster growth and mortality. This has effectively shortened tree life cycles^5^. Overall, tree mortality drivers acting from regional to local scales may include water stress or drought^6^, soil fertility^7^, wind disturbances^8, 9^, flooding^10, 11^, lightning^12, 13^, liana abundance and shading^2^. Human-related factors also affect mortality rates, such as forest degradation, fragmentation and edge effects^14, 15^. These drivers can kill the trees through the physiological mechanisms of carbon starvation and hydraulic failure, the interactions with biotic agents, and the modifications in plant structure that provoke stem breakage^2^. All this knowledge has been originated from field observations over a few hundred-forest sites in the Amazon, but we still lack tools for monitoring tree mortality over a large scale. To reduce the uncertainties of the mechanisms and accurately predict environmental and climate effects over tropical forests, remote sensing is an alternative for large-scale assessment of tree mortality^2, 16^. Nevertheless, the development of remote sensing methods for this purpose is still challenging, especially over the diverse and heterogeneous forests of the Amazon.

In principle, multi-temporal data from very high-resolution (VHR) satellites allow the estimation of tree mortality rates over tropical forests^17^. However, semi-automatic retrievals of tree mortality using VHR data are still challenging due to view-illumination effects. These effects alter the relative amounts of shadows and gaps viewed by the VHR sensors, which generally capture only the mortality of the tallest trees^18, 19^. In comparison with the VHR imagery, the multi-temporal data acquired by small-footprint airborne lidar instruments allow a more precise estimation of canopy gaps that can be related to tree loss at the canopy level^19–21^. On the other hand, standing dead trees do not necessarily generate gaps^18^ and airborne lidar mainly observes the upper-canopy trees in detail. As a result, the estimates of mortality from lidar canopy gaps are likely biased to some extent on observing tree mortality associated with broken and uprooted mode of death. In the Amazon, this mode of death represents approximately 39–55% of the total mortality but, on average, it is not significantly distinguished from the standing dead mode of death^22–24^.

The traditional gap concept consists of a ‘hole’ in the forest canopy extending through all levels down to an average height of 2 m above the ground^25^. A gap is created by the treefall of one or more trees, from either natural mortality or human-induced disturbances. There are two types of gaps reported in the literature^21^: (1) static gaps observed in a single-date lidar acquisition, showing the aggregated effects of gap opening and vegetation regeneration over time; and (2) dynamic gaps observed using two repeated lidar acquisitions, representing newly opened gaps from one date to another. However, the measurement of gaps is still challenging even using lidar. For instance, different gap definitions based on height cutoffs have provided different measurements of static gaps^21, 26, 27^. Alternatively, the height cutoff can be calibrated to a forest site based on the statistical analysis of the canopy height distribution^10^. However, this approach does not account for factors such as the presence of large open areas that are not necessarily treefall gaps. In temperate forests, another gap definition has been adopted considering the canopy relative height (RH) in relation to its neighborhood^28^. Nevertheless, this approach has not yet been tested over tropical forests.

The drivers of small-scale gap dynamics are also not completely understood. Local studies over Neotropical forests have shown that the topography, proxied by the Height Above the Nearest Drainage (HAND) index and terrain slope, can affect the gap dynamics with mechanisms related to wind exposure and waterlogged areas^10, 22, 23, 29^. Some forests showed gaps more frequently at valleys that increased in size toward the high terrain slopes^10, 23, 29^. Other forests did not show such relationships^22^. A study on small-scale gaps using Landsat satellite data have also pointed out for the effect of wind on opening gaps between 80 and 1000 m^2^^30^. Storms and lightning were also reported as the main causes of mortality in a forest at central Amazon^12^. Nevertheless, even considering these studies, not much is known regarding the variability of small-scale gaps along environmental and climate gradients across the Amazon region.

In this context, the analysis of lidar gap dynamics can significantly improve our comprehension on gap dynamics and tree mortality at a large scale in the Amazon. This is especially true given the new opportunities of increased lidar spatial coverage across the Brazilian Amazon. In 2016, 610 non-overlapping airborne lidar flight lines (300 m × 12.5 km) were obtained by the Improving Biomass Estimation Methods for the Amazon (EBA) project of the Brazilian National Institute for Space Research (INPE)^31^. Along with a few available multi-temporal lidar flight lines, such unique datasets can be used to evaluate drivers of gap dynamics and assess opportunities for correlations with field-measured tree mortality. The analysis of these datasets can bring new insights of forest dynamics patterns, drivers and mechanisms, and even guide the planning of new fieldwork activities for measuring tree mortality in the Amazon.

Here, we present the first large-scale study of lidar gap dynamics in the Amazon. The main goal was to provide a systematic assessment of canopy gaps across environmental-climate gradients and evaluate the possibilities of tree mortality estimates over tropical forests. Specifically, we aimed to answer the following research questions: (Q1) How are static and dynamic canopy gaps related in tropical forests and which gap definition best represents this relationship? (Q2) How do canopy gap dynamics derived from airborne lidar data vary across the Brazilian Amazon forests? (Q3) What landscape- and/or regional-scale factors drive gap variability in the Brazilian Amazon? (Q4) Does large-scale dynamic gaps estimates reproduce known spatial patterns of field-plot tree mortality estimates across the Amazon?

To answer Q1, we assessed the relationship between static and dynamic gaps using five multi-temporal airborne lidar datasets acquired over five test sites in Brazil with distinct forest types, vegetation structure and biomass. To answer Q2 and Q3, we evaluated the spatial variability of canopy gaps and some of the main drivers across the Brazilian Amazon. We used single-date airborne lidar data acquired in 610 non-overlapping lidar flight lines, representing a total sampled area of ~ 2300 km^2^. To answer Q4, we combined outputs from the previous analyses to estimate Amazon-wide dynamic gaps and related these estimates with tree mortality rates from long-term field inventory data.

## Results

### Multi-temporal lidar analysis of static and dynamic gaps relationship

The spatial matching between static and dynamic gaps showed variable responses amongst different combinations of methods and parameters explored on the five test sites (Sites-average *F1-Score (F)* from 0.01 to 0.51; detailed results in Supplementary Table S1). The RH delineation method, considering 50% maximum height on a 5-m window (W) neighborhood (*RH* = 50, *W* = 5), showed the best overall result (Sites-average: *F* = 0.51, *precision (p)* = 0.42, *recall (r)* = 0.66). When excluding the gaps pre-existing in the first lidar acquisition, an upper-bound performance estimate for this method indicated a modest increase in *p* and *F* (Sites-average: *F* = 0.62, *p* = 0.61, *r* = 0.66). The best result amongst the sites for the fixed height method was based on a 10-m height cutoff (Sites-average: *F* = 0.48, *p* = 0.47, *r* = 0.55), showing an inferior performance than the RH method.

**Figure 1:**
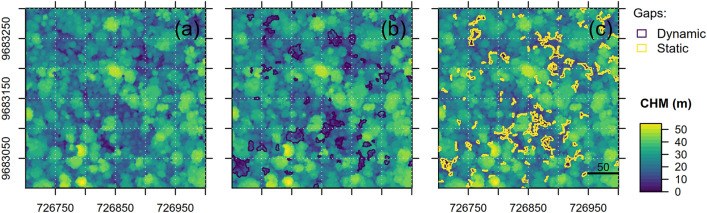
Example of the spatial match between canopy tree mortality (black) and gap delineation (yellow) based on relative height (*RH* = 50, *W* = 5) at the TAP site. The background is a Canopy Height Model (CHM) for the year 2012 in (**a**), and the year 2017 in (**b**) and (**c**). R v4.0.2 was used to plot this figure^32^.

The correspondence between static and dynamic gaps for the best method (*RH* = 50, *W* = 5) is illustrated in Fig. 1A–C. The dynamic gaps detected between 2012 and 2017 are shown in black (Fig. 1B) and the static gaps from 2017 data are represented in yellow (Fig. 1C) for a small subset of the TAP site. The method was able to map 68% of the dynamic gap areas (r_TAP_ = 0.68), with commission errors ranging from 34% (p_TAP_ = 0.66, upper-bound estimate) to 58% (p_TAP_ = 0.42, lower-bound estimate; Supplementary Table S1). For instance, in Fig. 1C, the top-right area showed examples of commission errors, i.e. delineated static gaps that do not correspond to dynamic gaps. Meanwhile, the bottom-middle area showed static gaps that successfully matched the dynamic gaps.

In a detailed inspection of the forest vertical structure prior to gap creation, we observed that static gaps detected dynamic gaps associated with trees below (< − 5 m, 98.5% accuracy) or close to the mean local canopy height (between − 5 and 5 m, 77% accuracy) with greater accuracy than that observed for trees above it (> 5 m, 37% accuracy) (Supplementary Figure S1). Correct detections below and close to the mean local canopy height represented 80.6% of the total dynamic gaps (15,582 out of 19,324 events).

**Figure 2:**
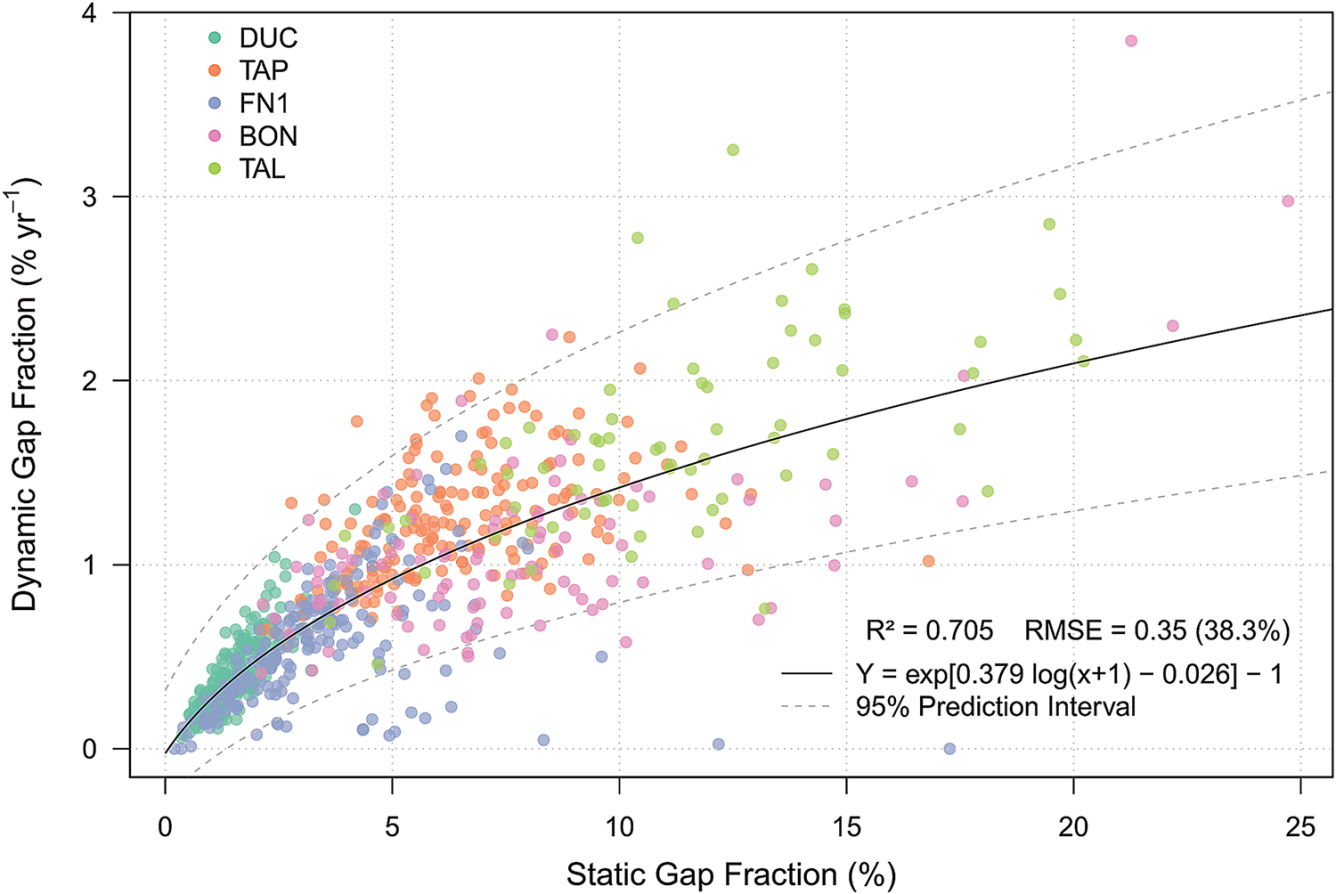
Relationship between static gap fraction (%) and annualized dynamic gap fraction (% year^−1^) for 5-ha plot areas (*n* = 780) over the five studied sites (DUC, TAP, FN1, BON, and TAL).

When we aggregated the gap detections to gap fraction at larger scales (5 ha plot area), we observed a significant, positive and non-linear logarithmic relationship between static gap fraction and annualized dynamic gap fraction (R^2^ = 0.705 ± 0.08, RMSE = 38.3 ± 13%; Fig. 2). The model residuals were randomly distributed. The uncertainty on estimating dynamic gap fraction from static gaps increased from small to higher static gap fraction values, as highlighted by the increasing prediction interval band. The sites with the highest mean static gap fraction estimates (mean ± SD%, *n* = number of samples), in decreasing order, corresponded to TAL (11.18 ± 3.97%, *n* = 69), BON (8.07 ± 4.28%, *n* = 95), TAP (6.4 ± 2.25%, *n* = 183), FN1 (3.3 ± 2.06%, *n* = 199), and DUC (1.47 ± 0.60%, *n* = 234).

### Single-date lidar gap spatial variability and drivers across the Brazilian amazon

#### Spatial variability of static gaps

**Figure 3:**
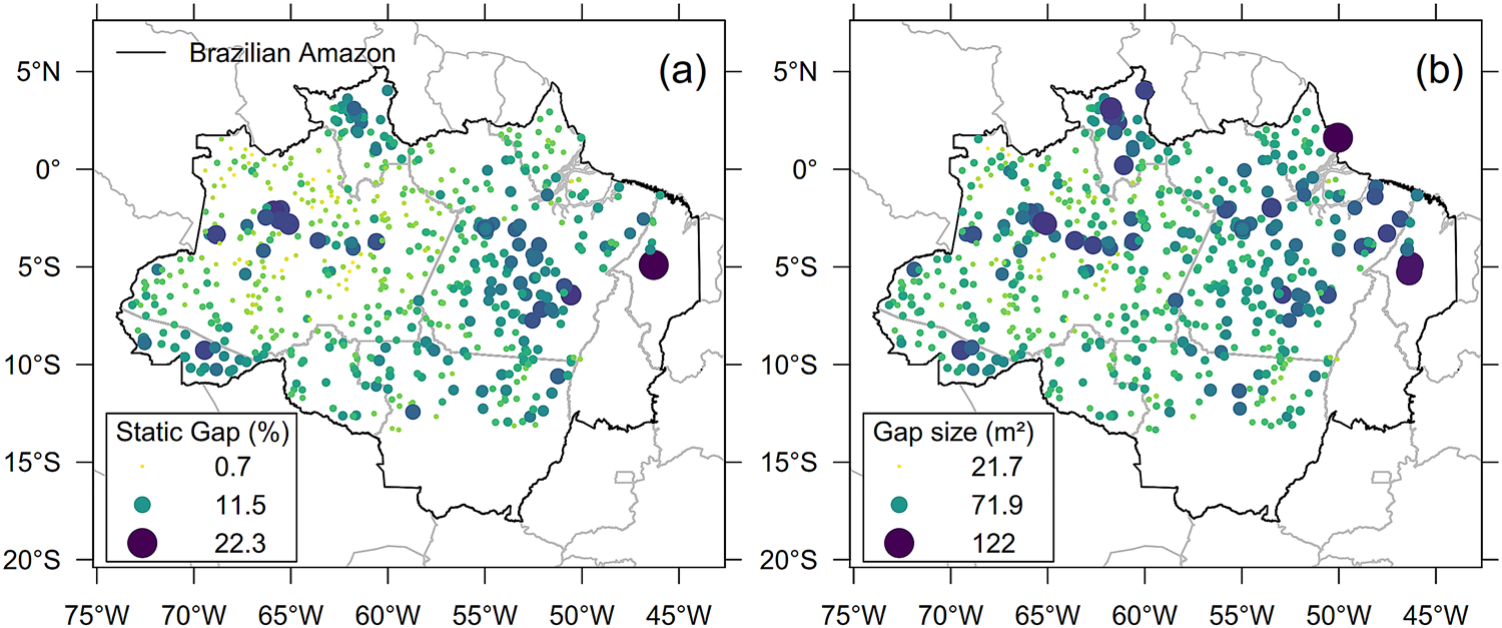
Canopy gaps based on the relative height (*RH* = 50, *W* = 5) across the Brazilian Amazon (*n* = 610 flight lines). (**a**) Gap fraction (%). (**b**) Mean gap size (m^2^).

The Brazilian Amazon region showed static gap fraction values ranging from 0.7 to 22.3% (Fig. 3A). The average gap fraction was 4.11%, but the majority of gap fraction values were lower than 5.17% (75th percentile). An overall lower gap fraction (Fig. 3A) was observed at central-east (mean ± SD: 3.57% ± 3.05) and north (3.68% ± 2) of the Amazon than at west (4.18% ± 2.13) and southeast (4.89% ± 2.47) regions of the study area described in Methods. High gap fraction values (> 5.17%) were found over the Brazilian states of Pará (~ 52° W; 5° S) and Acre (~ 69° W; 10° S). They were observed close to major water streams, such as the Amazonas and Madeira rivers, and to floodplains near the center of the Amazonas state (~ 65° W; 4° S). Besides these locations nearby rivers, predominantly lower gap fractions (< 2%) were found at the northwest Brazilian region over the Amazonas state. In comparison to other regions, the northwest has more intact forests, less water deficit and is expected to have increased AGB stocks. One site with anomalous high gap fraction (22.3%) was observed in eastern Amazon over the Arariboia indigenous land (~ 46° W; 5° S). This area has been degraded in recent years through illegal logging and fire. The gap fraction at the southeastern Xingu forests (~ 53° W; 12° S) ranged from 1.9 to 7.5%, with an average of 4.3%. This range of variability is likely lower than expected for one of the regions with most negative water deficit. The spatial variability of gap fraction (Fig. 3A) was consistent with the canopy height variability (Supplementary Figure S2). For instance, areas with high gap fraction showed the largest variation in canopy heights (CHM_SD_ from 8 to 14 m) and the lowest “minimum” height (CHM_P05_ < 10 m). The mean gap size (Fig. 3B) followed a similar spatial pattern as the gap fraction (Fig. 3A), with an average gap size across sites of 40.89 m^2^. Only a few sites showed larger mean gap sizes (> 100 m^2^).

#### Landscape- and regional-scale drivers of static canopy gaps

At the landscape-scale level, that is, within each lidar flight line, the HAND and slope variables were not able to explain gap occurrence or gap size (m^2^) variability (R^2^ ≈ 0). The same lack of relationship was observed by including/excluding the site as a random factor in the generalized linear models (GLM).

At the regional-scale level, that is, analyzing aggregated static gap fraction across sites, all tested variables were significantly correlated to gap fraction (*p* < 0.01) with absolute Pearson’s *r* values ranging from 0.21 to 0.46 (Supplementary Table S2). Seven variables were selected for modeling given their stronger correlation with gap fraction and lower correlation amongst themselves (*r* < 0.7): soil fertility proxied by Soil Cation Concentration *(SCC);* forest degradation proxied by non-forest distance *(Nonforest_dist)*; *Floodplains* cover; water deficit proxied by *Mean_def* and *SD_def*; and wind speed proxied by *Mean_vs* and *SD_vs.*

Three GLMs were empirically tested to estimate static gap fraction (Table 1, Supplementary Table S3): a full model with all seven variables (R^2^ = 0.557, RMSE = 52 ± 10%); a simplified model excluding wind variables and *SD_def* which were the least contributing variables (R^2^ = 0.523, RMSE = 53.3 ± 9.8%); and a final model based on the simplified model but including an interaction term between *Mean_def* and *Floodplains* (R^2^ = 0.57, RMSE = 44.9 ± 6%). All models showed randomly distributed residuals *versus* fitted values and were significantly different from a null model (*p* < 0.001). The final model achieved a superior gap fraction explanation than the full model and lower error, although not statistically different from each other (*p* = 1). Nevertheless, the final model showed a lower BIC value, indicating a better fit with more parsimony amongst the predictors and a lower multicollinearity (all VIF < 2). The final model residuals showed weak but significant spatial correlation effects (Moran’s I = 0.27, *p* < 0.01). However, the model parameter estimates and interpretations were not different from those from a model accounting for the spatial correlation (Supplementary Table S4).

**Table 1:** Estimated regression parameters (B), standard errors (SE B), t values (t) and *p* values for the generalized linear model (GLM) to estimate gap fraction.

Model variables	ΔR^2^	B	SE B	β	t	*P* value	VIF
(Intercept)	–	1.65	0.05	–	30.6	< 0.01	-
*SCC*	0.16	0.69	0.05	0.20	15.25	< 0.01	1.17
*Nonforest_dist*	0.03	− 0.13	0.02	− 0.09	− 6.3	< 0.01	1.39
*Floodplains*	0.17	1.63	0.10	0.08	15.75	< 0.01	1.94
*Mean_def*	0.09	0.03	0.002	0.12	11.15	< 0.01	1.68
*Floodplains:Mean_def*	0.05	− 0.11	0.01	− 0.12	− 8.39	< 0.01	1.61

Standardized beta coefficients (β), ΔR^2^ (change in R^2^ by adding the variable last to the model) and variance-inflation factors (VIF) for each predictor were also reported. Model achieved R^2^ of 0.57.

*SCC* soil cation concentration, *Nonforest_dist* distance to the nearest non-forest area, *Floodplains* floodplains cover fraction, *Mean_def* mean monthly water deficit.

**Figure 4:**
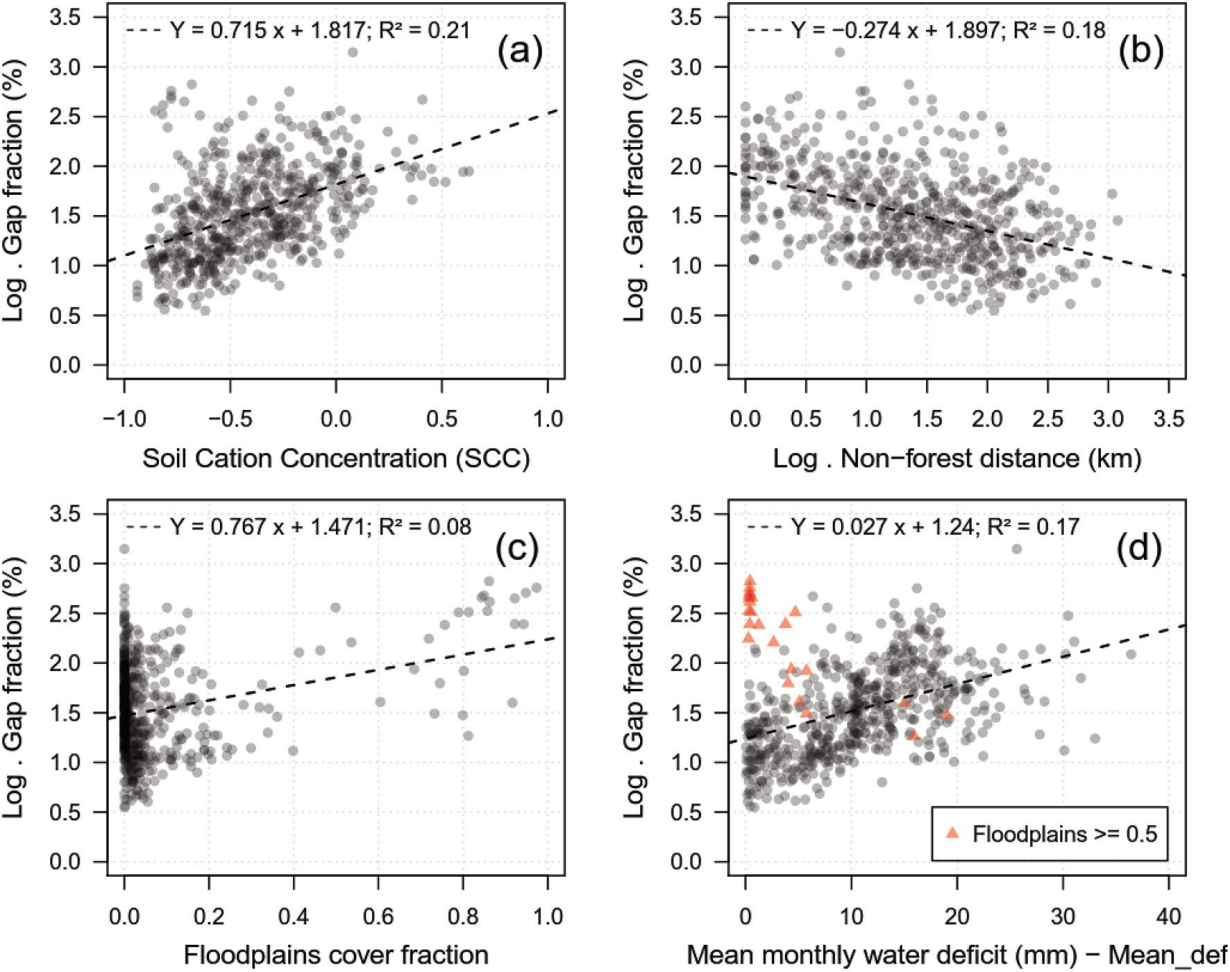
Relationships between gap fraction (log-scale) and predictors used in the final model. (**a**) Soil cation concentration (SCC). (**b**) Non-forest distance (km) (log-scale). (**c**) Floodplains cover fraction. (**d**) Mean monthly water deficit (mm)—*Mean_def*. Red triangles on panel (**d**) represent samples with floodplains cover fraction ≥ 0.5. The dashed line represents a linear model.

All predictors in the final model showed significant effects on gap fraction (*p* < 0.01) (Table 1). The predictors *SCC, Floodplains,* and *Mean_def* presented positive regression coefficients (B), indicating that an increase in these predictors caused an increase in gap fraction. In contrast, the *Nonforest_dist* and *Floodplains:Mean_def* had a negative B. These coefficient signs were corroborated by the expected relationship between these predictors and gap fraction (Fig. 4A–D). *Floodplains* and *SCC* solely explained the most gap fraction variability (ΔR^2^ = 0.17 and 0.16, respectively). The order of predictors’ importance ranked from the largest to the smallest absolute β values corresponding to: *SCC*, *Mean_def*, *Floodplains:Mean_def, Nonforest_dist*, and *Floodplains*. The *Floodplains* variable showed the lowest β, but part of its explained variability was also shared with the interaction term, thus it had more effect on gap fraction than *Nonforest_dist*. The interaction term (*Floodplains:Mean_def)* caused a significant improvement over the simplified model (Supplementary Table S3), likely associated with a better representation of areas with high gap fraction and close to zero water deficit areas, i.e. flooded or seasonally-flooded forests (red triangles in Fig. 4D).

#### Amazon-wide dynamic gaps map and opportunities for tree mortality estimates

**Figure 5:**
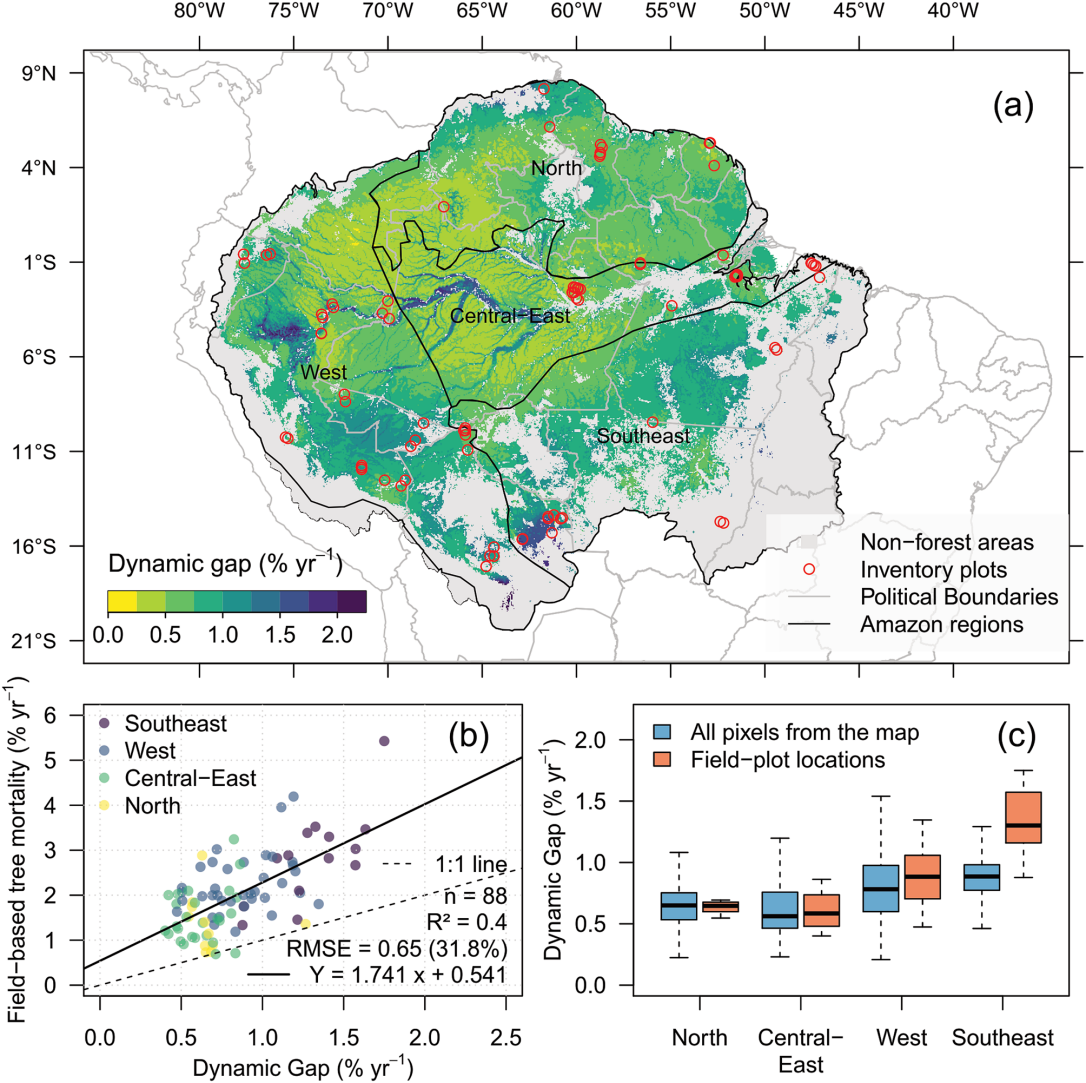
Large-scale estimates of dynamic gaps and relationship with tree mortality. (**a**) Dynamic gap (% year^−1^) generated at 5-km spatial resolution from the lidar static-dynamic gaps relationship (Fig. 3) and gap/environmental-climate model (Table 1). (**b**) Relationship between estimated dynamic gap fraction and field-based tree mortality from Brienen et al.^5^. (**c**) Estimated dynamic gap (% year^−1^) per Amazonian region using all values from the map (in blue, *n* > 40,000 pixels per boxplot) and just those corresponding to the field-plot locations (in orange, *n* = 88). The mean dynamic gap was statistically different between regions (*p* < 0.01). Dashed line represents the 1:1 line. R v4.0.2 was used to plot this figure^32^.

The dynamic gap fraction map showed diverse forest dynamics across the Amazon (Fig. 5A). Overall, lower dynamic gap fractions (< 0.5% year^−1^) were found at the undisturbed and wetter regions of the central-east, north and west Amazon. In contrast, higher dynamic gap fractions (> 1.5% year^−1^) were notably found over seasonally flooded forests, such as in the bottom-left of southeast Amazon (e.g. Noel Kempff Mercado National Park), west Amazon (e.g. Pacaya-Samiria National Reserve) and central-east Amazon (e.g. along Amazonas and Madeira rivers). Forests bordering the Andes at the southwest or in the transition zone with the Brazilian savannas (*Cerrado*) at the east also showed high dynamic gap fraction. These areas in the east have also a very high mean monthly water deficit (> 40 mm) (Supplementary Figure S4B). The spatialized SD of gap fraction estimates (Supplementary Figure S3B) indicated larger uncertainty over the southeast and west areas with higher dynamic gap fraction.

The dynamic gap fraction estimates were significantly associated with field-based stem tree mortality rates (R^2^ = 0.40, RMSE = 31.8% ± 9.2, Fig. 5B). However, it systematically underestimated the field mortality rates, as lidar dynamic gap fraction estimates were on average 58% lower than plot-based mortality rates. The results showed overall 20–35% higher annual dynamic gap fraction over the southeast (mean ± SD: 0.89% year^−1^ ± 0.2) and west (0.8% year^−1^ ± 0.28) regions than at the central-east (0.66% year^−1^ ± 0.28) and north (0.65% year^−1^ ± 0.17) regions (Fig. 5C). Considering all pixels over the Amazon in the analysis, we observed significant statistical differences in mean estimated dynamic gap fraction between the regions (ANOVA test; *p* < 0.01). This difference was more pronounced over the southeast Amazon, as deduced from the comparison of estimates using all pixels or just those pixels over the plot locations (blue and orange boxplots, respectively, in Fig. 5C). Most plots over the southeast region are located in forest fragments with fast dynamics, that is, in areas having potential high tree mortality and recruiting. When we considered only the field plot locations, the dynamic gap differences were also significant between regions (*p* < 0.01), except between central-east and north (*p* = 0.8).

## Discussion

We provided the first large-scale assessment of gap dynamics and its environmental-climate drivers across the Amazon forests. We also showed the opportunities for tree mortality estimates and monitoring based on the lidar gap dynamics. The dynamic gap fraction estimates notably matched the overall spatial patterns of stem tree mortality rates observed in the field plots, that is, the less-dynamic north and central-east regions in contrast to the more-dynamic west and southeast regions^1, 7, 24, 33, 34^. Additionally, our map indicated new findings of slower forest dynamics observed over large regions of undisturbed forests at the northwest (Amazonas state; 53° W; 12° S) and southeast Amazon (Xingu forests at the Mato Grosso state; 53° W; 12° S), as well as faster dynamics at eastern Amazon (near the center of Pará state; 54° W; 6° S). It also indicated high dynamic gaps for areas of bamboo-dominated forests at the southwest Amazon (70° W; 10° S), which had higher mortality rates than the average western forests^35^.

Part of this success was due to the use of the largest airborne lidar dataset already available over the Amazon (*n* = 610 flight lines). This unprecedented mission brought unique data over remote areas of the Amazon forest, where plot data collection was inexistent. Our dynamic gap estimates were about 60% lower in overall magnitude than the field-measured stem mortality rates. In addition to the uncertainties in the data analysis, the most probable explanation for the underestimation of tree mortality is the predominant detection of broken and uprooted mode of death associated with felled trees and the opening of gaps, which are ~ 50% of the total mortality^22–24^. Some standing dead trees also eventually break and fall, generating detectable gaps, but that rate is relatively low (4% year^−1^)^24^. Furthermore, the inevitable mismatch in scale between lidar and field inventory data certainly contributed to the observed differences in the magnitude of the estimates. The overall correlation could be partly associated to the gradient of forests dynamics across the regions, as forests in the west and southeast are known to have faster dynamics with increased tree mortality and productivity than the central-east and north regions^1^. Even though, their relationship was significant and relatively strong (R^2^ = 0.40) for a biological process observed at large scale, while only considering the spatial distribution of samples and not the temporal scale, and containing uncertainties in both measurements. The dynamic gaps (per unit area per unit time) are also likely correlated to AGB loss rate due to the predominant detection of upper-canopy trees loss. This relationship should be explored in further studies matching geo-located field plots with airborne lidar data.

We described a novel and significant relationship between static and dynamic gaps. We showed and quantified how static gaps information derived from single-date lidar could be used to estimate dynamic gaps, that is, structural changes in the forest canopy likely related to broken and uprooted trees. Hence, this finding opens venues for applications of gap metrics for forest monitoring beyond the simple representation of open spaces within the forest canopy. The developed method maps the gaps based on the relative height of 50% maximum height on a 5-m neighborhood, and explained 70% of annual dynamic gap fraction on the five tested sites. A potential explanation for the non-linearity on the static-dynamic gaps relationship is the gradient of canopy structure from more closed-canopy and/or undisturbed forests to more open-canopy and/or disturbed forests. In very dense forests, such as those from the DUC site, a static gap is highly correlated to newly created gaps from broken/uprooted mortality. This association is less strong for open forests, such as those observed at TAL, where gaps do not only represent mortality events but are also a part of the natural forest canopy structure. Hence, in order to offset this canopy structural gradient from closed to open and represent higher dynamic gap fractions (likely higher canopy turnover and mortality), an even higher static gap fraction is required.

When compared to the 10-m fixed height cutoff approach, the relative height method showed a similar but slightly stronger relationship with dynamic gap fraction and provided a dynamic adjustment to local canopy variability. This is very important given the high heterogeneity of the Amazon forest canopies. This results in a more stable and trustworthy gap delineation for large-scale applications in forests of varied canopy structure. In addition, the use of other fixed height cutoffs with lower values (2 and 5 m), traditionally adopted in tropical forests studies^21, 25, 29^, did not provide significant relationships between static and dynamic gaps. This means that the gaps detected using these thresholds are not directly related to recent canopy turnover events. Nevertheless, we expect that this finding contribute to the debate of gap delineation, where the relative height method should be an improvement over the fixed height cutoff approach. This is especially important if the intent is to represent recent structural changes in forests with varied canopy structure such as branch losses or tree losses associated to tree mortality or human disturbances.

Even considering the limitations to represent each natural and anthropogenic factor driving gap dynamics, our analysis detected water stress, soil fertility, floodplains and forest disturbance as key factors for predicting gap fraction at large scales. They explained up to 57% of its variability. The gap fraction followed a similar pattern to that of water stress in the Amazon (Supplementary Figure S4A,B), showing a gradient from the northwest (wetter) to the southeast (drier). The water stress had been previously associated with tree mortality measured in field plots and specifically to drought-related mortality^6, 36^.

Soil fertility is less often reported in the literature as a mortality driver^2^, probably due to the lack of soil data and/or perhaps because there are not many plots in regions with large soil nutrient gradients as depicted in the SCC product (Supplementary Figure S4C). In contrast, the lidar data here analyzed covered gradients from very low to high nutrient concentration. In the literature, higher soil fertility in younger alluvial soils of the western Amazon has been associated with faster forest dynamics, which, in turn, translates into higher tree mortality^7^. In our study, a few identified caveats about the SCC and its relationship with gap fraction include: (1) the representation of just a portion of soil fertility (Ca+Mg+K); (2) uncertainties related to the interpolation method; and (3) the soil sampling used to build the product, which was rather scarce in eastern Amazon^37^. These caveats should not be ignored because the high SCC in the eastern region was coincident with part of the highest gap fraction observations.

The observed patterns of floodplains and forest degradation (proxied by non-forest distance) (Supplementary Figure S4D) agreed with those of gap fraction. These results corroborate the literature on expected effects of increased gaps over water-logged lowlands^10, 11^ and tree mortality over flooded forests^38^. They corroborated also the increased mortality with degraded and/or fragmented forests^14, 15^. Further studies should assess with greater detail the gap variability with fragment edge effects, i.e. whether gap distribution significantly varies with distance from the fragment borders.

Our analysis did not detect the effects of wind on gap dynamics at the regional scale. However, wind has a well-known role in tropical forest mortality by uprooting trees, especially over the northwest Amazon^8, 9^. Moreover, its enhanced effect on snapping and uprooting trees that went through recent fire degradation has been demonstrated^39^. Our analysis does not show this effect probably because, at a regional scale, forests are likely adapted to the average wind variability. As a result, the mean gap fraction would not be driven by the mean wind speed. In addition, we show a much lower mean gap size (40.89 m^2^) than found in a study exploring wind mortality (360 m^2^)^30^. However, as reported in previous studies, extreme wind events could produce large forest disturbances, affecting areas even greater than 100 ha per event. Such large events are relatively rare^23^. Moreover, we did not find relationships of gap occurrence and size with slope and HAND topographic variables. The influence of topography on small-scale gaps over the relatively flat terrains of the Amazon is certainly less pronounced than that observed in other sloped forested areas of the world.

The influence of human-induced disturbances was likely underestimated in our analysis. The proximity of forests with high gap fractions to the ‘deforestation arch’ (agricultural frontier between savannas and tropical forests) confirmed some degree of human disturbance on results (e.g., logging, fire, fragmentation, edge effects). Because a reliable forest degradation product for the Amazon does not yet exist, we have attempted to assess this effect using the distance of each pixel to the nearest non-forest area (i.e. pastures, croplands, cities, roads, rivers). Although having a significant and negative relationship with gap fraction (i.e. the farther inside the forest, the less gaps), the non-forest distance metric did not explain much new of gap fraction variability when compared to other natural predictors. These disturbance effects probably consist of long-term processes (e.g., recovery from logging or fragmentation) rather than recent episodic events^15^. Fire disturbance was also not directly considered in our analysis of tree mortality. For instance, the TAL and BON sites were partially disturbed by fire before lidar data acquisition. However, the comparison of dynamic gaps in burned and non-burned areas mapped by^40^ within these sites did not show statistically significant differences between them (Supplementary Figure S5). Fire is widely known to affect tree mortality^41^. However, the effect of understory fires on tree mortality depends on fire frequency and severity.

Forest seasonality is another potential factor affecting gap fraction estimates from lidar data. In our study, the single-date lidar data were acquired almost regularly across the year, except for March. The acquisition covered both the rainy and dry seasons of the Amazon in 2016 (Supplementary Figure S6A). However, we did not find evidences of a seasonality control on gap fraction, that is, larger gap fractions were not more frequently observed in the dry season when compared to the rainy season (Supplementary Figure S6B). This was very likely related to the filtering of gaps based on size (minimum area of 10 m^2^) prior to gap fraction calculation, which eliminated potential small holes within crowns caused by leaf loss.

Our study points out for the importance of high-resolution lidar data to understand the relationships between gap dynamics and tree mortality. The acquisition of additional multi-temporal datasets as well as field data at poorly sampled regions in the Amazon are necessary. This would be extremely valuable to confirm patterns here observed and to reduce uncertainties in the analysis. Future directions of investigation include: (1) the use of additional multi-temporal lidar data (ideally annual) to confirm the magnitude of the relationships between static and dynamic gaps as well as between dynamic gaps and canopy mortality. Such relationships should consider the spatial and temporal scales, which may also vary across forest types and secondary successions; (2) the further investigation of drivers that might explain the remaining gap fraction variability, including effects of lightning, shading, and lianas^2, 12, 13^; (3) the exploration of orbital lidar data from the NASA’s GEDI (Global Ecosystem Dynamics Investigation) mission. While its footprint is too large to directly observe gaps, multiple overpasses of GEDI over the same location may capture variations in forest height associated with newly opened gaps; and (4) the development of new methods for mapping standing dead trees in tropical forests. Such approach can potentially leverage from the combined use of lidar and time series from optical instruments having high temporal resolution. These directions can guide future remote sensing studies and field campaigns aiming at a more complete understanding of the tree mortality patterns in the Amazon forests.

## Conclusions

1. Well-defined spatial patterns of forest dynamics were mapped over the Amazon leveraging the gap dynamics estimated from airborne lidar data. These patterns were notably consistent with field-based stem mortality rates. In contrast, they presented overall 60% lower rates probably due to the predominant detection of broken and uprooted trees.
2. Higher dynamic gap fractions were observed at the southeast (0.89% year^−1^) and west (0.8% year^−1^) regions when compared to the central-east (0.66% year^−1^) and north (0.65% year^−1^) regions. Areas previously not sampled in the ground now have some forest dynamics information to guide the development of future studies. Such areas include potential low tree mortality areas over undisturbed forests from the northwest and southeast regions of the Amazon, as well as high mortality areas over the eastern Amazon at the center of the Pará state.
3. Water stress, soil fertility, floodplains and forest degradation were found to be key predictors of gap dynamics at regional scale. A large portion of gap fraction variability still remains to be explained and explored in future studies.
4. The relative height method delineated static gaps more closely related to dynamic gaps than the fixed height cutoff approach. The static gaps detected by this method are able to explain 70% of annualized dynamic gap fraction variability with a logarithmic relationship. This means that the lidar gaps information are able to represent not only canopy openness, but also part of recent dynamics of canopy turnover and very likely tree mortality. The strength of correlation between dynamic gaps and canopy mortality across spatial and temporal scales is still to be determined in future studies using additional multi-temporal data.

## Methods

### Study area

**Figure 6:**
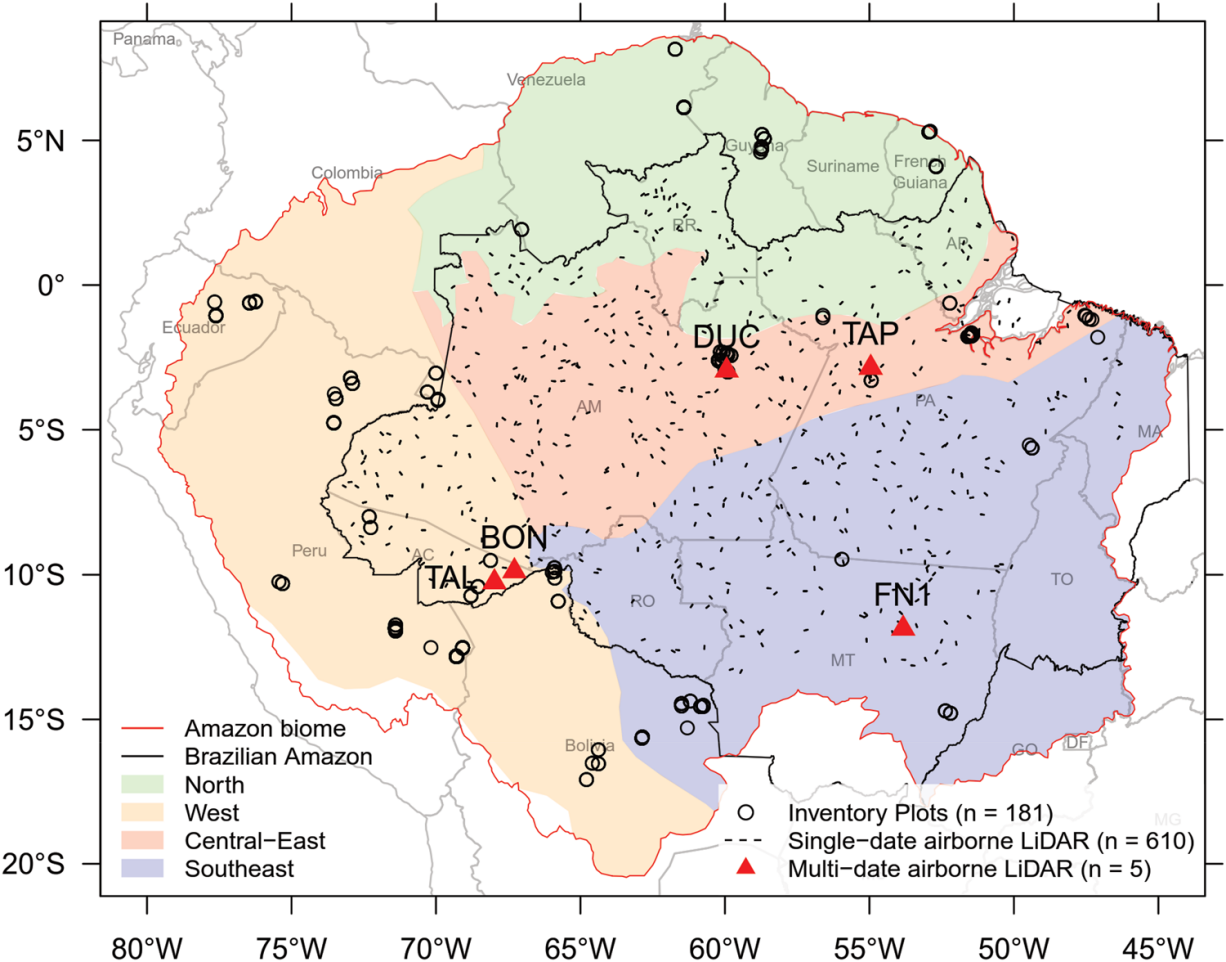
The Amazon in South America with colored regions, defined in Feldpausch et al.^33^, indicating faster (West and Southeast) and slower forest dynamics (Central-East and North). Small black lines represent single-date airborne lidar data acquisitions from the EBA project (*n* = 610 flight lines). Red triangles illustrate multi-temporal lidar data acquisition over five sites (BON, DUC, FN1, TAL and TAP). Circles indicate the location of field inventory plots (*n* = 181). R v4.0.2 was used to plot this figure^32^.

The study area was the Amazon basin (Fig. 6)^42^. The basin was sub-divided in four regions with markedly different forest dynamics, geography and substrate origin, adapted from the classification of Feldpausch et al.^33^: West (parts of Brazil, Colombia, Ecuador and Peru), Southeast (Bolivia and Brazil), Central-East (Brazil) and North (Brazil, Guyana, French Guiana and Venezuela). The natural vegetation mainly corresponds to broadleaf moist forests and tropical seasonal forests, with both terra firme and seasonally flooded forests. Across the Amazon, there is a wide range of average monthly rainfall (100–300 mm) and dry season length (DSL) (0–8 months)^43^.

The five sites selected for the multi-temporal assessment of the static and dynamic gaps relationship (red triangles in Fig. 6) were: Adolpho Ducke forest (DUC), Tapajós National Forest (TAP), Feliz Natal (FN1), Bonal (BON) and Talismã (TAL). These areas were chosen to represent distinct forest types, vegetation structure and biomass stocks. The predominant vegetation types consisted of dense rain forests (DUC and TAP), seasonal forests (FN1), and open rain forests (TAL and BON). DUC and FN1 are mostly undisturbed forests, while TAP underwent fire and/or selective logging in the past. TAL and BON were affected by a known fire occurrence in 2010. The sites cover a gradient of aboveground biomass (AGB) that increase, in average, from TAL (185 Mg ha^−1^), FN1 (235 Mg ha^−1^), BON (251 Mg ha^−1^), and DUC (327 Mg ha^−1^) to TAP (364 Mg ha^−1^)^44^.

### Data acquisition and pre-processing

#### Airborne lidar data

Multi-temporal lidar data were obtained by an airplane at each of the five sites (red triangles in Fig. 6), as part of the Sustainable Landscapes Brazil project. The time-interval window was close to 5 years and was sufficient to measure the long-term aggregated dynamics of tree mortality. The area covered by lidar in the 2012–2018 period was ~ 43 km^2^, ranging from 480 ha at TAL site to 1200 ha at DUC site (Supplementary Table S5).

In addition to the multi-temporal datasets, 610 single-date airborne discrete-return lidar data strips (approx. 300 m wide by 12.5 km long; ~ 3.75 km^2^ each) were acquired during 2016 (acquisition dates in Supplementary Figure S6A) using the Trimble HARRIER 68i system at an airplane. The average flight height was 600 m above ground and the scan angle was 45° (dataset from the EBA project^31^).

For both lidar datasets, multiple lidar returns were recorded with a minimum point density of 4 points m^−2^. Horizontal and vertical accuracy ranging from 0.035 to 0.185 m and from 0.07 to 0.33 m, respectively.

Following the procedures described by Dalagnol et al.^19^, the lidar point clouds were processed into canopy height models (CHM) of 1-m spatial resolution. The steps of CHM processing included the: (a) classification of the points between ground and vegetation using the lasground, lasheight, and lasclassify functions from the LAStools 3.1.1^45^; (b) creation of a Digital Terrain Model (DTM) using the TINSurfaceCreate function from FUSION/LDV 3.6^46^; (c) normalization of the point cloud height to height above ground using the DTM; and (d) CHM generation by extracting the highest height of vegetation using the CanopyModel function from FUSION.

#### Environmental and climate data

To analyze the environmental and climatic drivers of gap dynamics, we used a spatialized set of variables and products for the whole Amazon, including: (a) HAND product at 30 × 30 m^47^; (b) slope calculated from the Shuttle Radar Topography Mission (SRTM) at 30 × 30 m^48^; (c) soil fertility proxied by SCC at 11 × 11 km^37^; (d) floodplain cover map at 30 × 30 m^49^; (e) forest degradation proxied by a non-forest distance map derived from the 30-m global forest change dataset v1.4 (2000–2016)^50^; (f) monthly mean rainfall (mm), climate water deficit (mm) and wind speed (m s^−1^), obtained from the TerraClimate dataset at 5 × 5 km (1958–2015)^43^; and (g) DSL at 28 × 28 km^51^. All variables and products, except HAND and slope, were resampled to the predominant spatial resolution of most datasets (5 km × 5 km), especially the climate data. We used the SRTM instead of the lidar DTM because the very narrow lidar DTMs (300–500 m) would not permit to determining the lowest point in the terrain to accurately calculate the HAND for every pixel.

#### Long-term field inventory data

We used data from 181 long-term field inventory plots from the RAINFOR network (Fig. 6)^5^. The data were collected at closed canopy mixed forests with vegetation structure preserved from fire and logging. All trees with diameter at breast height (DBH) ≥ 10 cm were measured at least twice^5^. These plots had 852 censuses from 1975 to 2013 with median plot size of 1 ha. The mean re-census interval was 3 years. Tree stem mortality rates (*m*; % year^−1^) were calculated as the coefficient of exponential mortality for each census interval and each plot^52^ (Eq. 1). The *m* estimates were then averaged by plot and were weighted by the censuses interval length, in years^1^.

1 $$m = \left[ {ln\left( {N0} \right) - ln\left( {Nt} \right)} \right]/t$$

where *N0* and *Nt* are the initial and final number of trees, and *t* is the censuses interval.

### Data analysis

#### Gap definition and static–dynamic gaps relationship

Dynamic gaps were detected using multi-date lidar data at the five study sites: DUC, TAP, FN1, BON, and TAL. We define here dynamic gaps as those opened between two periods of observation associated with canopy turnover events, including tree mortality. For this purpose, we calculated a delta height difference of 10 m between the two acquisitions (~ 5 years apart) and filtered for detections with area greater than 10 m^2^. This height difference was strongly correlated with tree loss at the canopy level in previous studies^19, 20^. Because standing dead trees do not necessarily generate gaps, we assume that the dynamic gaps are mostly related to the felled canopy trees associated with broken and uprooted mode of death.

Static gaps were delineated using the CHM from the second lidar acquisition at the five sites (Supplementary Material S1). We applied and compared two types of gap delineation: a traditional method based on a fixed height cutoff (H = 2, 5 or 10 m), and an alternative method based on the relative height (RH = 33, 50, and 66% maximum tree height) around a neighborhood (W = 5–45 m). Since the relative height method did not depend on absolute height values, it should better account for local canopy variability and lower stature vegetation, as opposed to the fixed height method. For both methods, we tested a variety of parameters in the search of an optimal calibration amongst the sites. We filtered gaps for a minimum area of 10 m^2^, which corresponded to an approximation of the mean canopy area of trees greater than 5-cm DBH in tropical forests^21^. We also filtered them for a maximum area of 1 ha to automatically exclude open areas that very likely did not correspond to small-scale disturbance from treefall gaps^21^.

The spatial match between each static and dynamic gap event was assessed by intersecting the detections and calculating metrics of precision (*p*), recall (*r*) and F1-score (*F*) (Eqs. 2–4) (more information at Supplementary Material S1). *p* represents the percentage of total correct detections, *r* represents the percentage of reference data correctly mapped, and *F* represents the harmonic mean between *p* and *r*, that is, a balance between commission and omission errors. Methods and parameters were compared to determine the optimal method for static gap delineation, i.e. higher *F* means greater agreement between static and dynamic gaps.

2 $$Precision\left( p \right) = true \, positives/number \, of \, gap \, polygons$$

3 $$Recall\left( r \right) = true \, positives/number \, of \, mortality \, polygons$$

4 $$F1 - score\left( F \right) = \left( {2 \times p \times r} \right)/\left( {p + r} \right)$$

Finally, considering the optimal gap delineation method, we modeled the relationship between static-dynamic gaps at the landscape scale using a linear regression. For this purpose, annualized dynamic gap fraction and static gap fraction (i.e., the area occupied by gaps in relation to the total area of the flight line) were calculated at the 5-ha scale. Following the strategy by Wagner et al.^53^, we defined this value after several simulation tests between variable estimates, change rates and plot area (Supplementary Figure S7). Data and residuals were inspected for normality, and variables were transformed to the logarithmic scale prior to the linear model fitting. To assess the model, we calculated the coefficient of determination (R^2^), absolute Root Mean Square Error (RMSE) and relative RMSE (%) (ratio of RMSE and the mean of observations). To obtain more reliable and unbiased estimates of the model predictive performance, we calculated the RMSE considering out-of-sample values with a leave-one-site-out cross-validation (CV) strategy. Thus, we fitted the model with four sites and calculated the RMSE with predicted and observed values for the site not used in the modeling. We repeated this process for all five sites. A 95% prediction interval described the variability of tree mortality estimates from the gap fraction.

#### Spatial variability of static gaps across the Brazilian Amazon

We delineated static gaps on the single-date airborne lidar datasets (*n* = 610 flight lines) using the optimal gap delineation method and parameters assessed in the previous section. To characterize the gaps variability across the region, we calculated the gap fraction and mean gap size for each site.

#### Assessment of landscape- and regional-scale drivers of static canopy gaps

To quantify the relationship between static gaps and landscape- and regional-scale predictors, we employed correlation matrices and generalized linear models (GLM). Binomial GLM and Gaussian GLM were applied for landscape and regional models, respectively (detailed information at Supplementary Material S2). Models were assessed using a tenfold CV approach with 30 repetitions. The gap data used in this analysis were those obtained from the 610 single-date lidar data. We defined landscape-scale drivers as those showing great heterogeneity intra-site such as the topography (HAND and slope variables). We defined regional-scale drivers as those having great variability across sites such as the rainfall (Mean_pr and SD_pr), wind speed (Mean_vs and SD_vs), climate water deficit (Mean_def and SD_def), DSL, SCC, floodplains, and non-forest distance.

Through the modeling we evaluated if gap occurrence (presence or absence) and gap size increased at valleys and steep terrains of the Amazon, represented by low HAND and high slope, respectively. As previously demonstrated with tree mortality ground observations, we also tested if gap fraction would increase with: (1) higher water stress, represented by low Mean_pr, and high SD_pr, Mean_def, SD_def, and DSL; (2) higher soil fertility, expressed by high SCC; (3) higher wind speed, proxied by high Mean_vs and SD_vs; (4) higher forest degradation/fragmentation, represented by low non-forest distance; and (5) areas of seasonally flooded forests, expressed by high floodplains cover. Model residuals were inspected in comparison to fitted values using also variogram and Moran’s I analyses to assess for potential biases and spatial correlation (detailed information in Supplementary Material S2). Static gap fraction and *Nonforest_dist* were transformed to log-scale due to non-normality data.

#### Amazon-wide dynamic gaps mapping and relationship with tree mortality

To obtain a map of dynamic gap estimates over the Amazon, we first applied the GLM model based on environmental and climate drivers to estimate static gap fractions for the whole region. We then applied the static–dynamic gaps relationship to estimate annualized dynamic gap fraction (% year^−1^). To explore the opportunities for tree mortality estimates based on gap dynamics, we compared the spatialized dynamic gap estimates with time-averaged mortality rates from long-term field inventory data using a linear model. The model was assessed using a tenfold CV approach with 30 repetitions and the RMSE calculated out-of-sample. We acknowledge that the comparison between field tree mortality and lidar gap estimates is not trivial. However, it is the best source available of independent mortality data to compare the results. Field plot-estimates located within the same 5-km cell of the lidar gap estimates were averaged, resulting in 88 pairs of lidar- and field-estimates samples for validation. The mean annualized dynamic gap fraction per Amazonian region (Fig. 6) was extracted and compared using one-way ANOVA and post-hoc Tukey–Kramer tests.

## Supplementary Information

Supplementary Information

## Data Availability

The data frames used in the analysis as well as the dynamic gap fraction map are available at the Zenodo repository:doi: https://doi.org/10.5281/zenodo.4262542. Raw multi-date airborne lidar data can be obtained at https://www.paisagenslidar.cnptia.embrapa.br/webgis. Raw single-date airborne lidar data are not currently openly available because of legal contractual reasons associated with the Improving Biomass Estimation Methods for the Amazon (EBA) project. These data will be freely available in 2022.
