# Supplementary material for: Large-scale variations in the dynamics of Amazon forest canopy gaps from airborne lidar data and opportunities for tree mortality estimates
Source: Sci Rep. 2021 Jan 14;11:1388. doi: 10.1038/s41598-020-80809-w (PMC7809196; doi:10.1038/s41598-020-80809-w)
Supplement: Supplementary file 1 — Supplementary Information [file 41598_2020_80809_MOESM1_ESM.docx]

**Supplementary Information for**

**Large-scale variations in the dynamics of Amazon forest canopy gaps from airborne lidar data and opportunities for tree mortality estimates**

Ricardo Dalagnol^1*^, Fabien H. Wagner^1,2^, Lênio S. Galvão^1^, Annia S. Streher^1^, Oliver L. Phillips^3^, Emanuel Gloor^3^, Thomas A. M. Pugh^4,5^, Jean P. H. B. Ometto^6^, Luiz E. O. C. Aragão^1,7^

^1^ Remote Sensing Division, National Institute for Space Research - INPE, São José dos Campos, 12227-010, SP, Brazil

^2^ GeoProcessing Division, Foundation for Science, Technology and Space Applications - FUNCATE, São José dos Campos, 12210-131, SP, Brazil

^3^ School of Geography, University of Leeds, Leeds LS2 9JT, UK

^4^ School of Geography, Earth and Environmental Sciences, University of Birmingham, Birmingham, B15 2TT, UK

^5^ Birmingham Institute of Forest Research, University of Birmingham, Birmingham, B15 2TT, UK

^6^ Earth System Sciences Center, National Institute for Space Research - INPE, São José dos Campos, 12227-010, SP, Brazil

^7^ Geography, College of Life and Environmental Sciences, University of Exeter, Exeter EX4 4RJ, UK

*Corresponding author. E-mail address: ricds@hotmail.com (R. Dalagnol).

**This PDF file includes:**

Methods S1 and S2

Figures S1 to S8

Tables S1 to S5

**Methods**

**Gap delineation (S1)**

The first gap definition was based on the classical concept of Brokaw et al. [1] and a height cutoff H. All pixels with height below the H cutoff were considered as a gap. We tested different H cutoff parameters (H = 2, 5, 10 m) covering values commonly used in studies of Neotropical forests [2-5]. The fixed height gap delineation was performed using the ForestGapR R-package [6]. The second gap definition was based on the canopy RH in comparison to the maximum height within a neighborhood of size W [7]. The assumption behind this method is that a tree mortality event does not necessarily create a gap that extends all the way to the ground. It can create a hole in the canopy with a relative height below that of neighboring trees. The original W and RH parameters [7] consisted of 10 m and 66% of max. height, respectively. We tested this method by varying window size parameter (W = 5, 9, 15, 25, 35, 45 m) and relative heights (RH = 33, 50 and 66% of max. height). The W range to represent gaps was defined to reach up to very large tree crowns (45 m). The RH range was empirically chosen to represent heights below a threshold that would consist in a gap. To better delineate gap centers, we also included a fixed H cutoff of 2 m, based on the traditional gap definition [1].

To assess each method and parameters, the spatial match between static and dynamic gaps was assessed by intersecting the gap delineation with the mortality detection and by calculating metrics of precision (*p*), recall (*r*) and F1-score (*F*) (Eq. 1-3). *F* represents the harmonic mean between the precision and recall, that is, a balance between commission and omission errors, where higher *F* values would represent a greater agreement between gaps and mortality. True positives were defined as individual static gap delineations that intersected dynamic gap detections with at least 25% intersection. This criterion was established in order to ensure confidence in results. Simply considering an intersection as successful could overestimate the overall performance. We acknowledge that the dynamic-static gap relationship may be affected by vegetation regrowth (closing gaps and resultant omission error) or represent previous mortality to the first lidar acquisition (commission error). Following the assumption that pre-existent gaps in the first CHM date may also represent mortality, we also calculated the performance excluding pre-existent gaps for the best method and parameters. Therefore, we defined an upper-bound performance estimate correcting for the unobserved mortality and a lower-bound estimate.

*Precision (p) = true positives / number of gap polygons* (1)

*Recall (r) = true positives / number of mortality polygons* (2)

*F1-score (F) = (2 * p * r) / (p + r)* (3)

To find the optimal gap delineation method amongst sites, the performance between the five study sites was averaged (“sites-average”) and assessed. The gap detection was further analyzed as a function of tree height of the first lidar acquisition (prior to the gap opening) in relation to the mean local canopy height (radius of 50 m around the gap detection).

**Drivers modeling (S2)**

Generalized linear models (GLM) were built using the R-packages *stats* [8] and *lme4* [9]. For landscape-scale drivers (Height Above the Nearest Drainage - HAND and slope variables), we tested whether the variables (30 x 30 m spatial resolution) could predict individual gap occurrence and gap size distribution. For this purpose, we extracted the variables from pixels with gap presence and absence. For the gap absence, we randomly selected pixels at non-gap covered areas (same number of gap presence). We fitted a GLM with binomial family, namely a logistic regression, to predict the probability of gap occurrence (Eq. 4). We also fitted a GLM with Gaussian distribution to predict gap size distribution (Eq. 5). We tested the models considering the site as a random effect and the predictors individually or combined.

$logit\left( {GapProb}_{i} \right)= \ln\left( \frac{p}{1-p} \right)=\beta_{0}+\beta_{j}X_{ij}+ u_{j}Z_{ij}+ \varepsilon_{i}$ *(*4)

where $GapProb$*_i_* is the gap probability for pixel *i*, *β_0_* is the intercept term, *β_j_* is the regression coefficient of predictor *j*; *X_ij_* and *Z_ij_* are the predictor matrices for fixed-effect model parameters *β_j_* and random-effect parameters *u_j_*, respectively; *ε_i_* is the residual error assumed to follow a normal distribution.

${GapSize}_{i}= \beta_{0}+\beta_{j}X_{ij}+ u_{j}Z_{ij}+ \varepsilon_{i}$ *(*5)

where *GapSize_i_* is the gap size for pixel *i*, and the remaining terms are the same as Eq. 4.

For regional-scale drivers (Mean_pr and SD_pr, Mean_vs, SD_vs, Mean_def, SD_def, DSL, SCC, floodplains, and non-forest distance), we employed a Gaussian GLM (similar to Eq. 5) to quantify how much of the variability of gap fraction (dependent variable) was explained by the regional-scale variables (independent variables). On the other hand, the gap fraction showed clear spatial patterns and a much more intuitive interpretation. To reduce the effects of multicollinearity, we selected variables based on an exploratory analysis using a correlation matrix. A Gamma GLM model was also tested showing similar but weaker predictive power (out-of-sample RMSE = 45.5 ± 6%) than the Gaussian GLM (RMSE = 44.9 ± 6%).

The models were compared based on their explanatory power considering the R² and Bayesian Information Criterion (BIC) [10]. The multicollinearity of predictors within the model was assessed by the variance-inflation factors (VIF), which indicates the degree of interaction/correlation between independent variables (high values = high inter-correlation).

To assess the model for spatial autocorrelation effects, we calculated the Moran’s I index using the final model residuals (out-of-sample errors) and considering the nearest eight neighbors. To explore up to what distance the samples would show correlation, we employed a variogram analysis. This distance was determined to be 100 km (Supplementary Figure S8). To address the spatial dependence, we conducted a distance-based random sampling scheme to select non-correlated samples (>100 km afar from each other) and fit the model. This procedure was repeated 30 times with different samples (average of 172 samples from the 610 total samples). We performed out-of-sample 10-fold cross-validation.


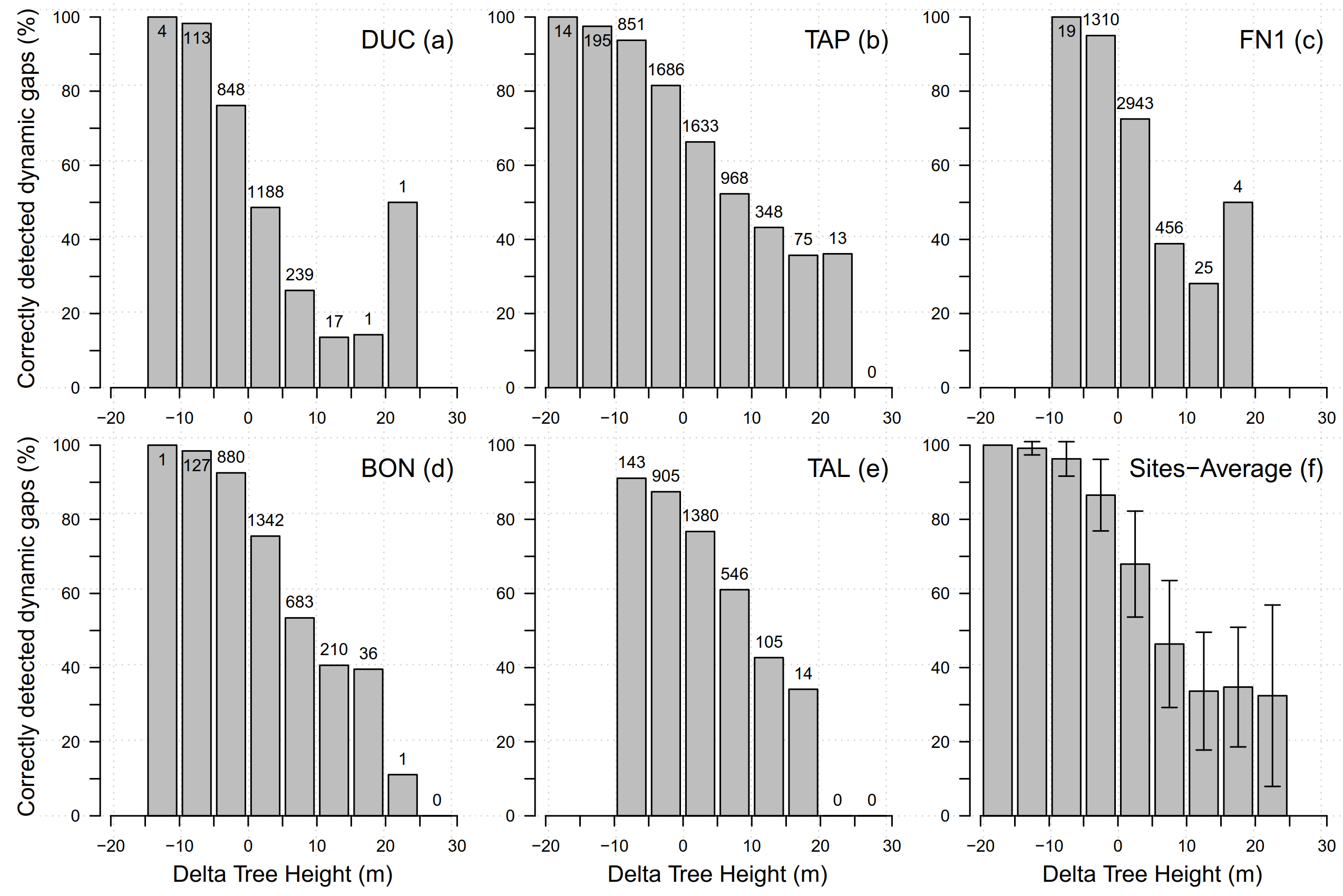


**Figure S1.** Percentage of correctly detected dynamic gaps by delineated static gaps (Relative height method, *RH*=50, *W*=5), as a function of the relative tree height classes for the sites. (**a**) DUC. (**b**) TAP. (**c**) FN1. (**d**) BON. (**e**) TAL. Results considering all sites (“sites-average”) are shown in (**f**). The numbers close to the bars represent the absolute detections for that delta tree-height class. The 95% confidence interval, based on *t*-value score, is indicated in (**f**) for the sites-average.


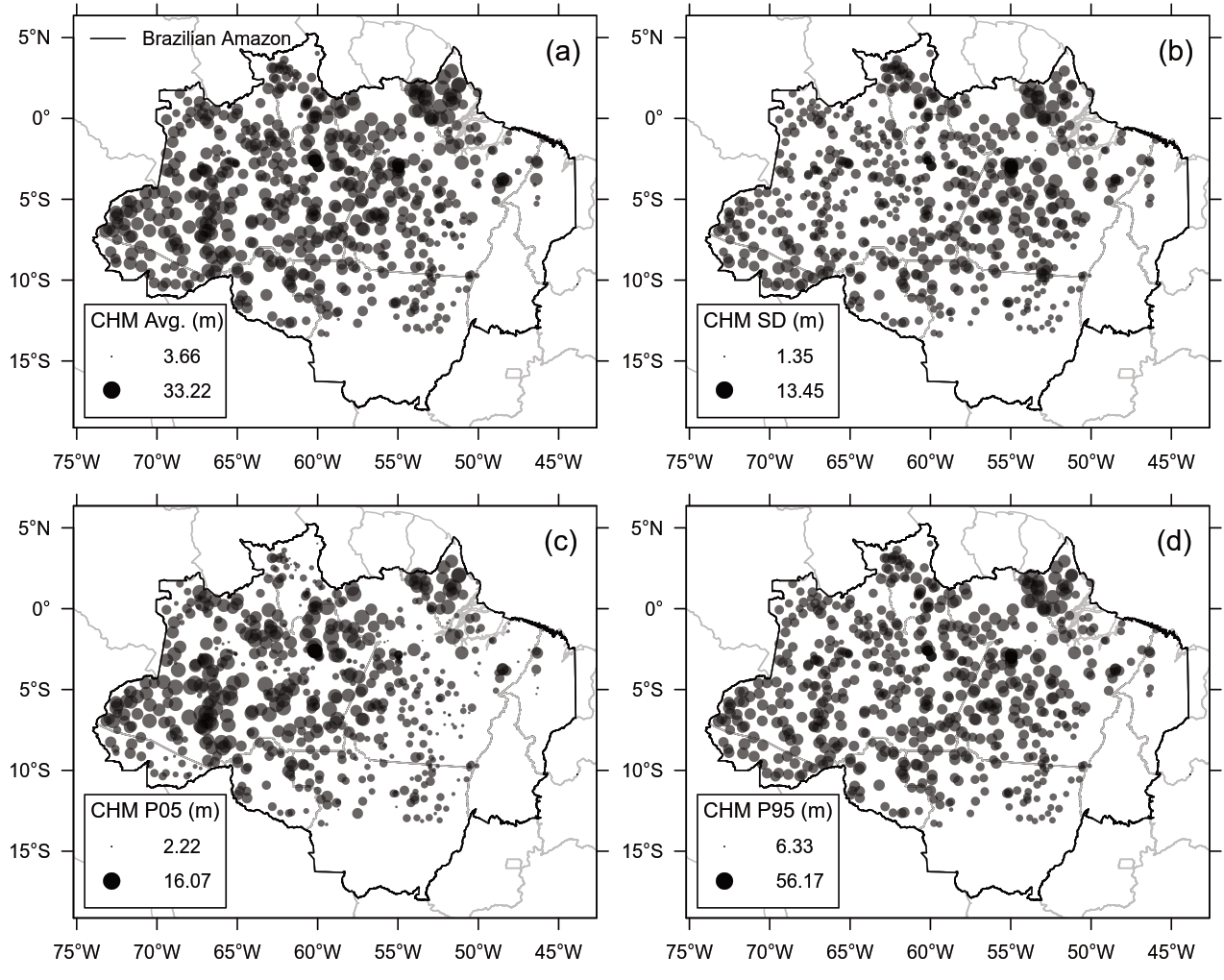


**Figure S2.** Spatial variability of canopy height model (CHM) across the Brazilian Amazon (*n* = 610 flight lines). (**a**) CHM average (CHM_AVG_). (**b**) CHM standard deviation (CHM_SD_). (**c**) 5^th^ percentile (CHM_P05_). (**d**) 95^th^ percentile (CHM_P95_). Units represent meters.


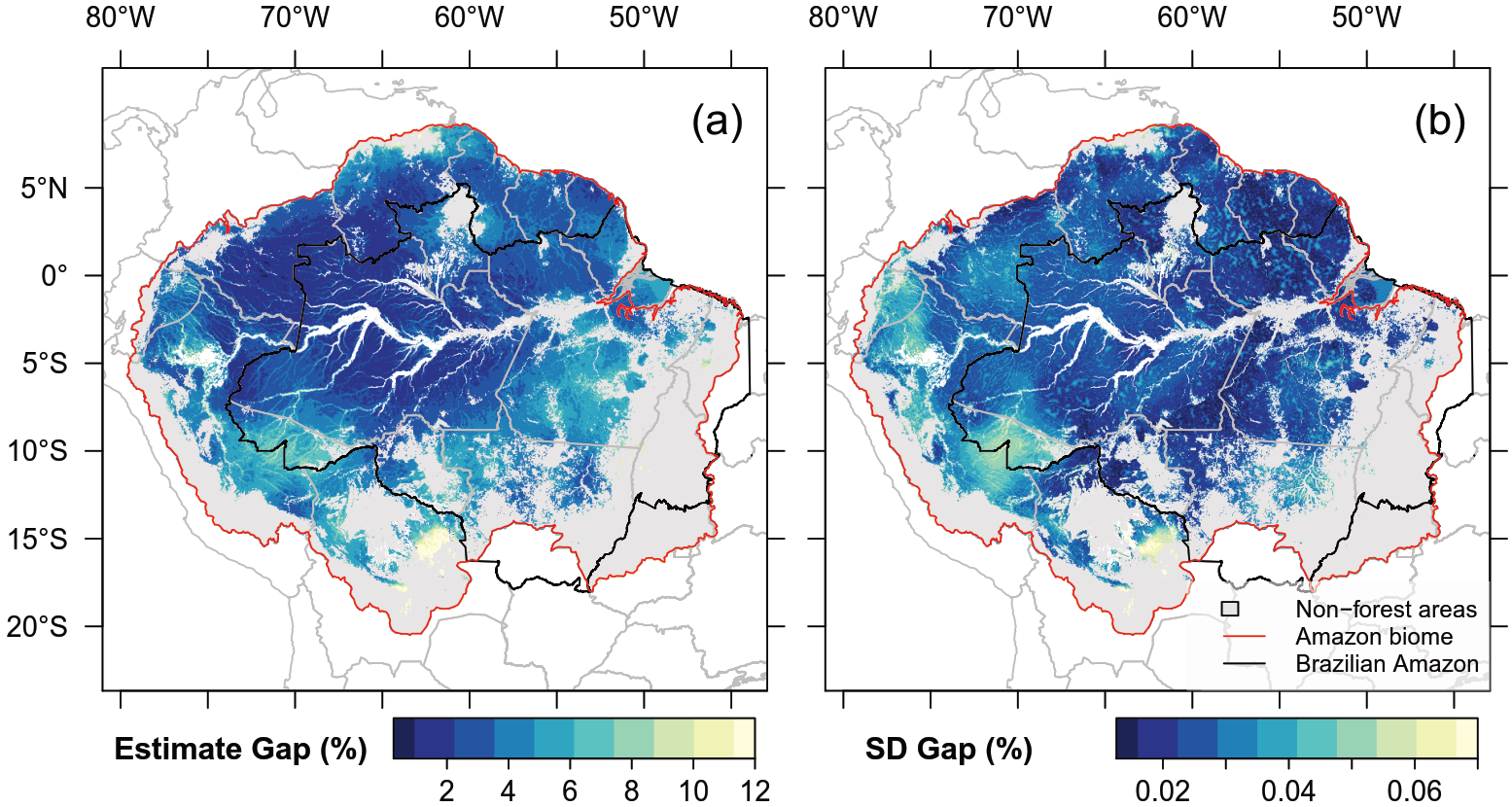


**Figure S3.** Amazon-wide static gap fraction estimates based on environmental and climate drivers. (**a**) Estimate static gap fraction (%). (**b**) Standard deviation (SD) of static gap fraction estimate (%). Areas in white correspond to either missing data or rivers. R v4.0.2 was used to plot this figure [15].


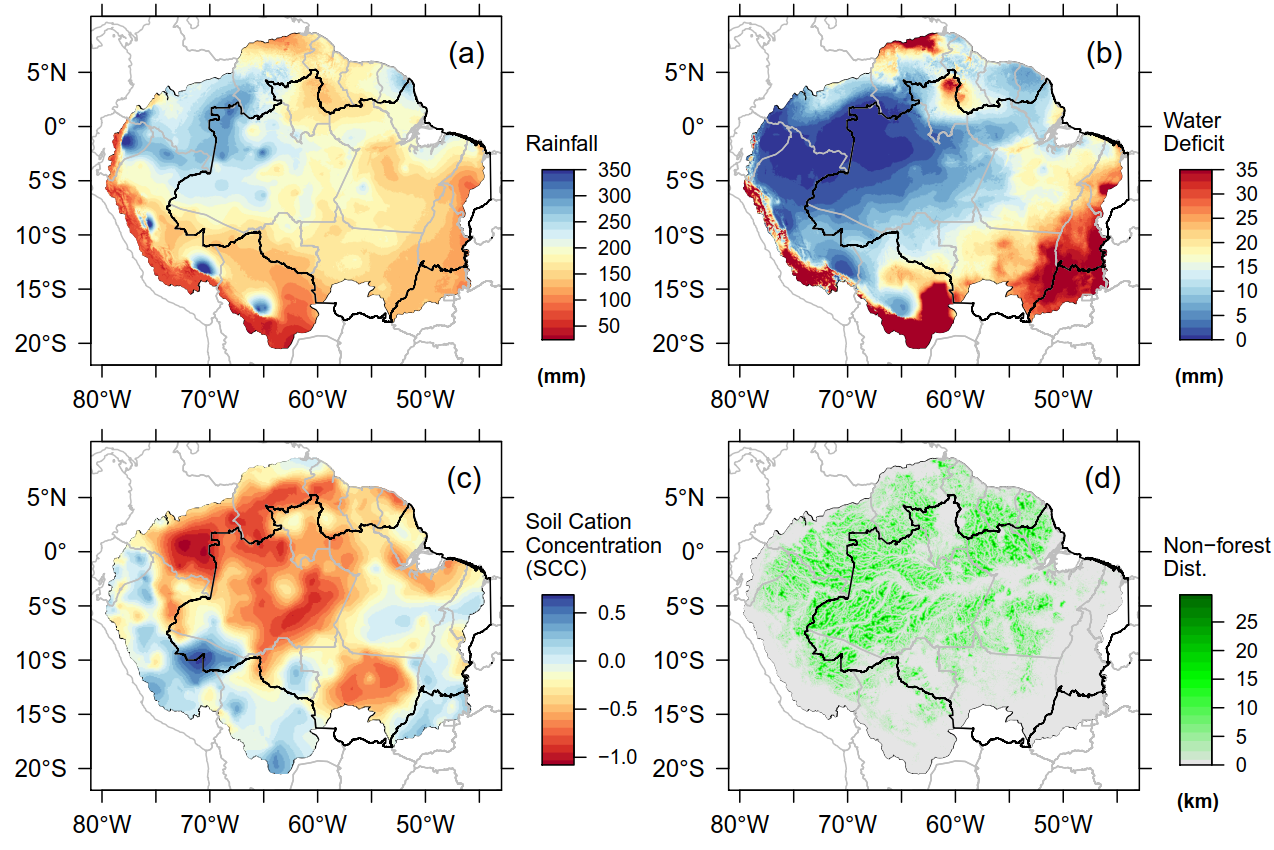


**Figure S4.** Environmental and climate variables for the Amazon forests. (**a**) Average monthly rainfall (mm). (**b**) Average monthly water deficit (mm) from the TerraClimate product [11]. (**c**) Soil Cation Concentration (SCC) [12], a proxy for soil fertility. (**d**) Non-forest distance (km) derived from the Global Forest Cover product [13], a proxy for forest degradation. R v4.0.2 was used to plot this figure [15].


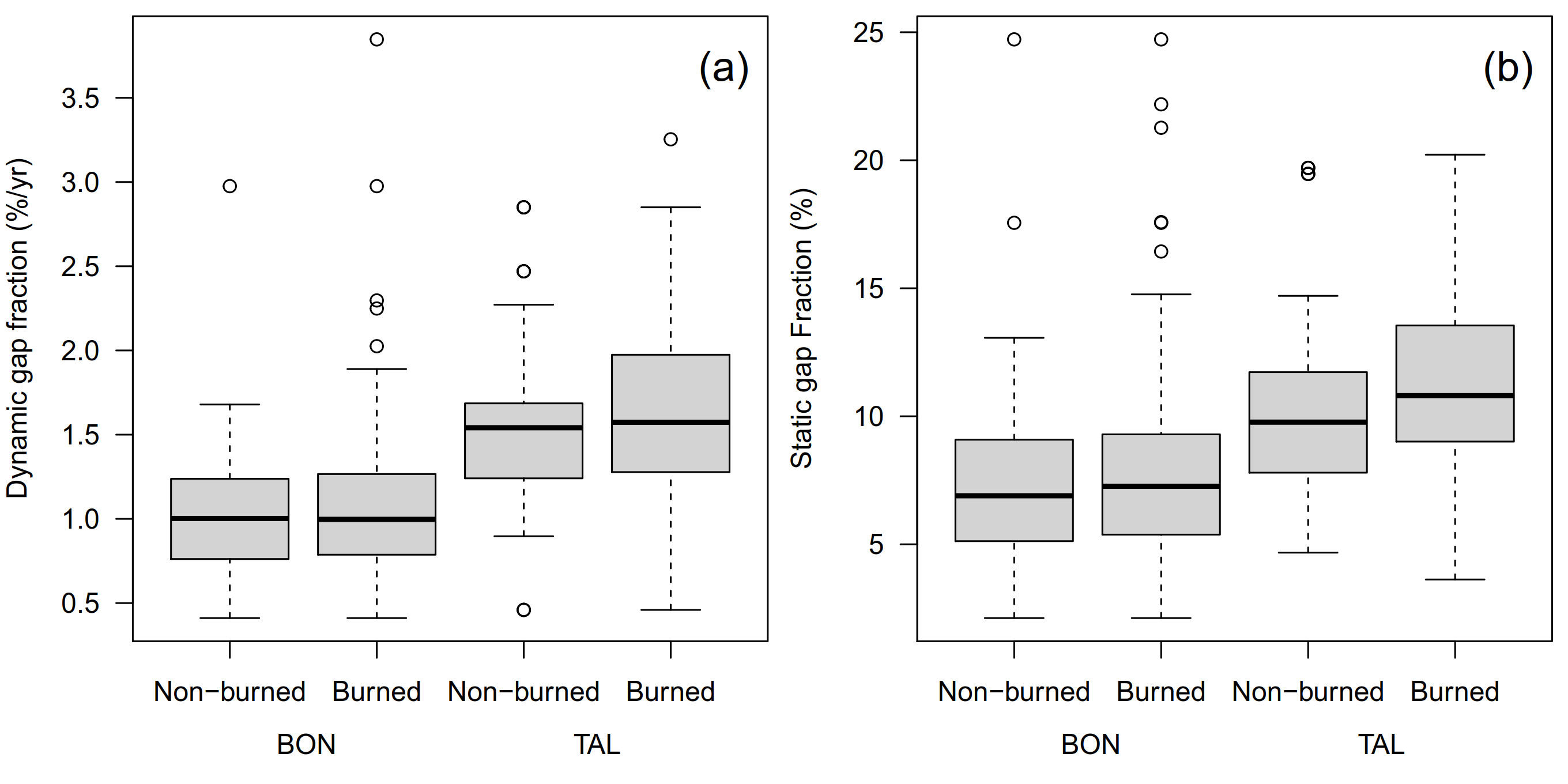


**Figure S5.** Dynamic and static gaps in burned forests (BON and TAL). (**a**) Annual dynamic gap fraction (% yr^-1^). (**b**) Static gap fraction (%) for burned and non-burned forests at the BON (*n* = 95) and TAL (*n* = 69) sites. Burned areas were derived from Sato [14]. Data correspond to aggregated values at 5-ha.


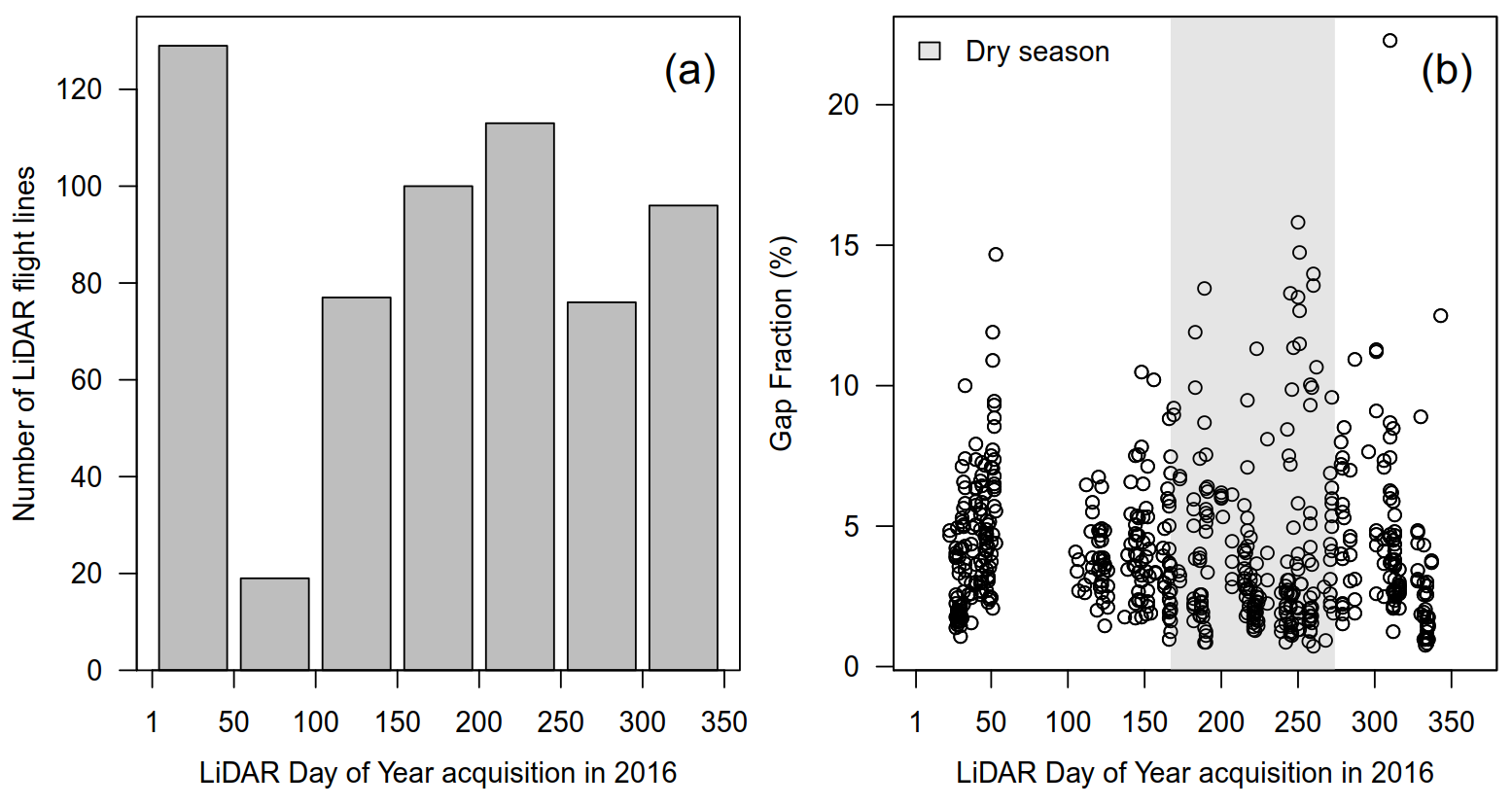


**Figure S6.** Single-date airborne lidar Day of Year acquisition in 2016 for the 610 flight lines from the EBA project. (**a**) Frequency of lidar data acquisition per date. (**b**) Variations in lidar static gap fractions in the rainy and dry seasons of the Amazon region.


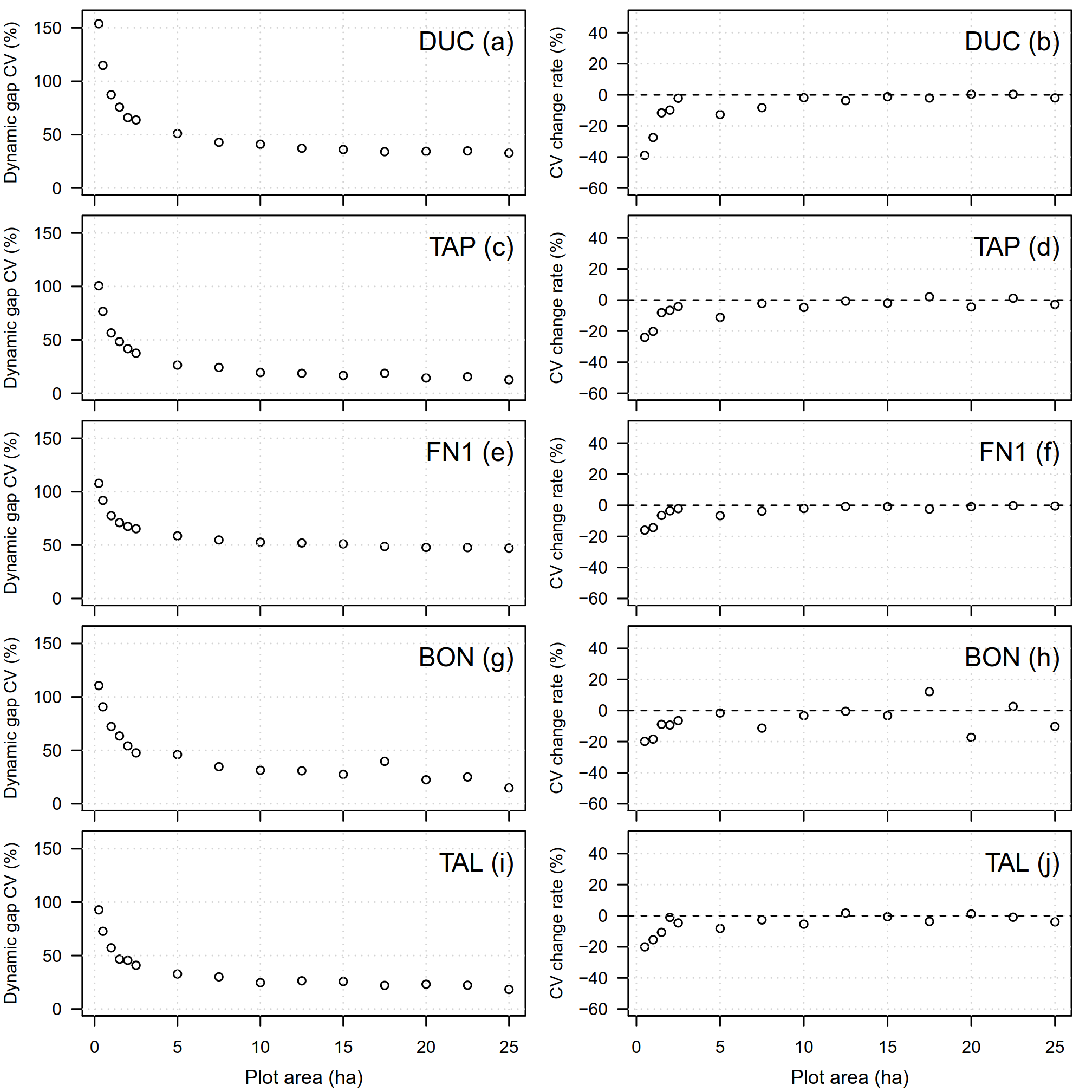


**Figure S7.** Variability of lidar dynamic gap fraction with plot area for five Amazonian sites. The dynamic gap coefficient of variation (CV, %) and the CV change rate are shown for (**a-b**) DUC. (**c-d**) TAP. (**e-f**) FN1. (**g-h**) BON. (**i-j**) TAL. Stable estimates of dynamic gap fraction are obtained at a minimum 5-ha plot area.


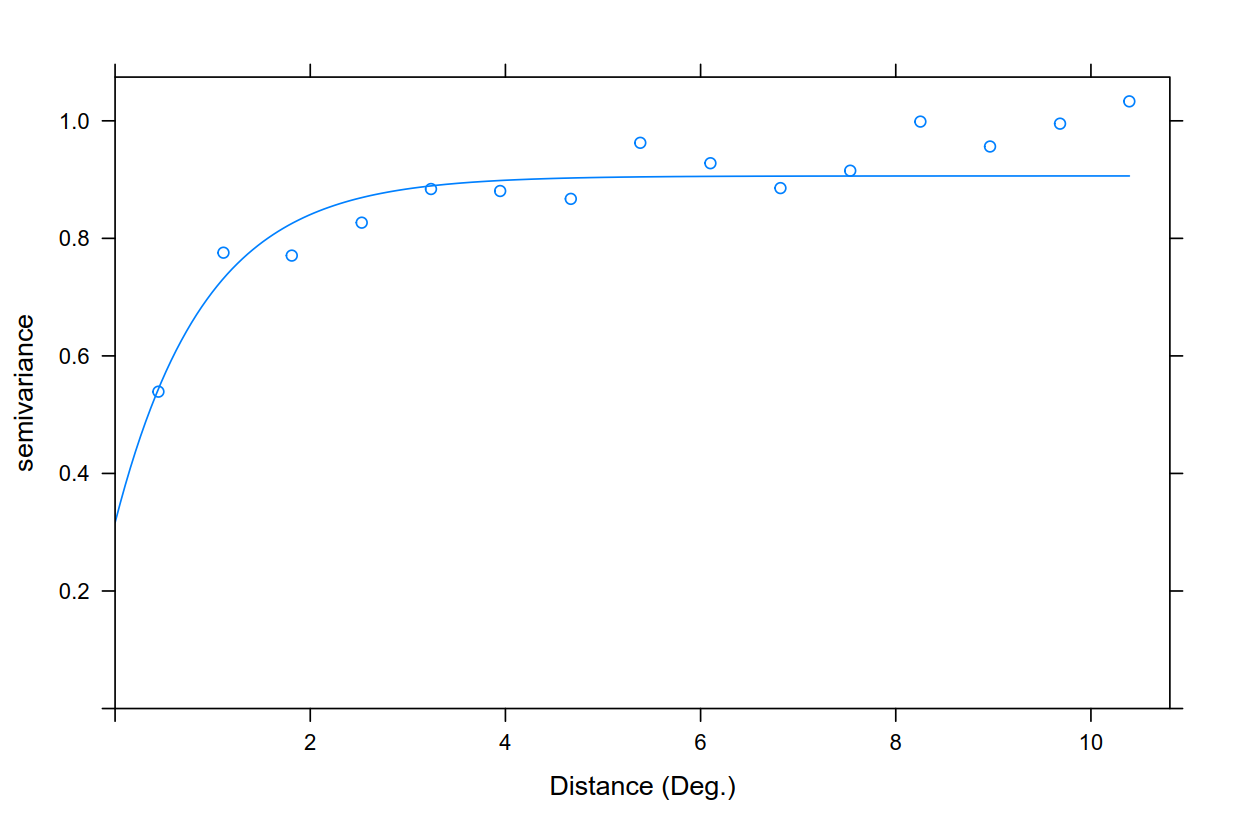


**Figure S8.** Variogram of 610 static gap fraction values to assess the spatial autocorrelation distance.

**Table S1.** Performance of different static gap definitions to represent dynamic gap fraction for each site and an average between sites (“sites-average”). Methods and parameters are ranked by the Sites-Average *F* metric. Method’s parameters include: *W* = window size (meters); *RH* = percentage of maximum height in the neighborhood W cutoff; and *H* = fixed height cutoff. Performance metrics include: *p* = precision; *r* = recall; and *F* = F1-score. Values of *p*, *r*, and *F* range from 0 to 1, with larger values indicating better performance.

| **Method and parameters** | **DUC** | | | **TAP** | | | **FN1** | | | **BON** | | | **TAL** | | | **Sites-Average** | | |
| --- | --- | --- | --- | --- | --- | --- | --- | --- | --- | --- | --- | --- | --- | --- | --- | --- | --- | --- |
|  | ***p*** | ***r*** | ***F*** | ***p*** | ***r*** | ***F*** | ***p*** | ***r*** | ***F*** | ***p*** | ***r*** | ***F*** | ***p*** | ***r*** | ***F*** | ***p*** | ***r*** | ***F*** |
| Variable: W=5, RH=50 | 0.48 | 0.51 | 0.49 | 0.42 | 0.68 | 0.52 | 0.49 | 0.71 | 0.58 | 0.35 | 0.69 | 0.46 | 0.37 | 0.74 | 0.5 | 0.42 | 0.66 | 0.51 |
| Variable: W=15, RH =33 | 0.56 | 0.37 | 0.45 | 0.45 | 0.58 | 0.51 | 0.59 | 0.54 | 0.57 | 0.38 | 0.61 | 0.47 | 0.41 | 0.66 | 0.51 | 0.48 | 0.55 | 0.5 |
| Variable: W=25, RH =33 | 0.51 | 0.43 | 0.47 | 0.42 | 0.65 | 0.51 | 0.53 | 0.57 | 0.55 | 0.35 | 0.67 | 0.46 | 0.39 | 0.66 | 0.49 | 0.44 | 0.6 | 0.5 |
| Variable: W=35, RH =33 | 0.48 | 0.47 | 0.47 | 0.4 | 0.68 | 0.51 | 0.5 | 0.58 | 0.53 | 0.33 | 0.69 | 0.44 | 0.37 | 0.63 | 0.47 | 0.42 | 0.61 | 0.49 |
| Fixed: H=10 | 0.59 | 0.34 | 0.43 | 0.53 | 0.44 | 0.48 | 0.43 | 0.75 | 0.54 | 0.37 | 0.62 | 0.46 | 0.41 | 0.62 | 0.5 | 0.47 | 0.55 | 0.48 |
| Variable: W=45, RH =33 | 0.45 | 0.49 | 0.47 | 0.39 | 0.69 | 0.5 | 0.48 | 0.56 | 0.52 | 0.32 | 0.7 | 0.44 | 0.36 | 0.6 | 0.45 | 0.4 | 0.61 | 0.48 |
| Variable: W=9, RH =33 | 0.65 | 0.29 | 0.4 | 0.5 | 0.48 | 0.49 | 0.66 | 0.46 | 0.54 | 0.42 | 0.52 | 0.46 | 0.41 | 0.59 | 0.48 | 0.53 | 0.47 | 0.47 |
| Variable: W=9, RH =50 | 0.34 | 0.73 | 0.46 | 0.33 | 0.84 | 0.47 | 0.36 | 0.87 | 0.51 | 0.26 | 0.83 | 0.4 | 0.3 | 0.72 | 0.43 | 0.32 | 0.8 | 0.45 |
| Variable: W=15, RH =50 | 0.27 | 0.81 | 0.41 | 0.29 | 0.81 | 0.43 | 0.3 | 0.89 | 0.44 | 0.22 | 0.81 | 0.35 | 0.26 | 0.55 | 0.35 | 0.27 | 0.78 | 0.4 |
| Variable: W=5, RH =66 | 0.22 | 0.88 | 0.35 | 0.26 | 0.92 | 0.41 | 0.23 | 0.96 | 0.37 | 0.2 | 0.84 | 0.32 | 0.22 | 0.57 | 0.32 | 0.23 | 0.83 | 0.35 |
| Variable: W=25, RH =50 | 0.23 | 0.85 | 0.36 | 0.26 | 0.65 | 0.37 | 0.24 | 0.85 | 0.38 | 0.18 | 0.68 | 0.29 | 0.23 | 0.43 | 0.3 | 0.23 | 0.69 | 0.34 |
| Variable: W=5, RH =33 | 0.79 | 0.12 | 0.21 | 0.6 | 0.24 | 0.35 | 0.75 | 0.23 | 0.35 | 0.46 | 0.29 | 0.35 | 0.42 | 0.31 | 0.36 | 0.6 | 0.24 | 0.32 |
| Variable: W=35, RH =50 | 0.2 | 0.87 | 0.33 | 0.25 | 0.55 | 0.34 | 0.21 | 0.81 | 0.34 | 0.17 | 0.54 | 0.26 | 0.21 | 0.34 | 0.26 | 0.21 | 0.62 | 0.3 |
| Variable: W=45, RH =50 | 0.18 | 0.88 | 0.31 | 0.24 | 0.48 | 0.32 | 0.19 | 0.76 | 0.31 | 0.16 | 0.4 | 0.22 | 0.2 | 0.27 | 0.23 | 0.19 | 0.56 | 0.28 |
| Variable: W=9, RH =66 | 0.13 | 0.97 | 0.23 | 0.2 | 0.66 | 0.31 | 0.13 | 0.96 | 0.23 | 0.12 | 0.57 | 0.2 | 0.14 | 0.33 | 0.2 | 0.15 | 0.7 | 0.23 |
| Fixed: H=5 | 0.89 | 0.03 | 0.06 | 0.68 | 0.11 | 0.19 | 0.74 | 0.18 | 0.29 | 0.46 | 0.21 | 0.28 | 0.4 | 0.22 | 0.29 | 0.63 | 0.15 | 0.22 |
| Variable: W=15, RH =66 | 0.09 | 0.98 | 0.17 | 0.16 | 0.35 | 0.22 | 0.08 | 0.86 | 0.15 | 0.1 | 0.14 | 0.12 | 0.12 | 0.13 | 0.12 | 0.11 | 0.49 | 0.15 |
| Variable: W=25, RH =66 | 0.06 | 0.89 | 0.12 | 0.15 | 0.16 | 0.15 | 0.06 | 0.55 | 0.11 | 0.1 | 0.04 | 0.05 | 0.09 | 0.05 | 0.06 | 0.09 | 0.34 | 0.1 |
| Variable: W=35, RH =66 | 0.05 | 0.7 | 0.1 | 0.15 | 0.09 | 0.11 | 0.06 | 0.27 | 0.09 | 0.11 | 0.02 | 0.04 | 0.09 | 0.03 | 0.04 | 0.09 | 0.22 | 0.08 |
| Variable: W=45, RH =66 | 0.05 | 0.51 | 0.09 | 0.14 | 0.05 | 0.07 | 0.05 | 0.16 | 0.08 | 0.12 | 0.02 | 0.03 | 0.09 | 0.02 | 0.03 | 0.09 | 0.15 | 0.06 |
| Fixed: H=2 | 0.98 | 0.01 | 0.01 | 0.69 | 0.01 | 0.02 | 0.67 | 0.01 | 0.01 | 0.56 | 0.01 | 0.02 | 0.35 | 0.01 | 0.02 | 0.65 | 0.01 | 0.01 |

**Table S2.** Pearson’s correlation (*r*) between static gap fraction and predictors. All correlations were significant at 1% statistical level, except for the cells containing ‘*ns*’.

| **Variables** | **Gap Frac.** | **SCC** | ***NF_dist*** | ***Mean_def*** | **DSL** | ***SD_vs*** | ***Mean_pr*** | ***SD_def*** | **Floodpl.** | ***SD_pr*** | ***Mean_vs*** |
| --- | --- | --- | --- | --- | --- | --- | --- | --- | --- | --- | --- |
| **Gap Frac.** | 1 |  |  |  |  |  |  |  |  |  |  |
| **SCC** | 0.46 | 1 |  |  |  |  |  |  |  |  |  |
| ***NF_dist*** | -0.43 | *ns* | 1 |  |  |  |  |  |  |  |  |
| ***Mean_def*** | 0.42 | 0.35 | -0.37 | 1 |  |  |  |  |  |  |  |
| **DSL** | 0.41 | 0.46 | -0.26 | 0.87 | 1 |  |  |  |  |  |  |
| ***SD_vs*** | 0.38 | 0.28 | -0.25 | 0.78 | 0.62 | 1 |  |  |  |  |  |
| ***Mean_pr*** | -0.38 | -0.46 | 0.24 | -0.78 | -0.87 | -0.49 | 1 |  |  |  |  |
| ***SD_def*** | 0.36 | 0.34 | -0.34 | 0.97 | 0.86 | 0.75 | -0.73 | 1 |  |  |  |
| **Floodpl.** | 0.27 | -0.23 | -0.26 | -0.27 | -0.35 | -0.22 | 0.3 | -0.28 | 1 |  |  |
| ***SD_pr*** | 0.24 | 0.23 | -0.23 | 0.74 | 0.77 | 0.55 | -0.51 | 0.79 | -0.32 | 1 |  |
| ***Mean_vs*** | 0.21 | 0.11 | -0.28 | 0.55 | 0.45 | 0.74 | -0.3 | 0.54 | -0.11 | 0.38 | 1 |

SCC = soil cation concentration; Floodpl. = floodplains fraction; *NF_dist* = non-forest distance; *Mean*_*def* and *SD_Def* = mean and standard deviation monthly water deficit; *Mean_vs* and *SD_vs* = mean and standard deviation monthly wind speed; DSL = dry season length; *Mean_pr* and *SD_pr* = mean and standard deviation monthly precipitation.

**Table S3.** Estimated regression parameters (B), standard errors (SE B), t values (t) and *p*-values for the Generalized Linear Model (GLM) to estimate gap fraction. Standardized beta coefficients (β), ΔR² (change in R² by adding the variable last to the model) and variance-inflation factors (VIF) for each predictor were also reported.

| Model | ΔR² | B | SE B | β | t | p-value | VIF |
| --- | --- | --- | --- | --- | --- | --- | --- |
| Full model | **R² = 0.557; BIC = 334.6** | | | | | | |
| (Intercept) | - | 1.9 | 0.09 | - | 21.5 | < 0.01 | - |
| *SCC* | 0.16 | 0.68 | 0.05 | 0.20 | 14.6 | < 0.01 | 1.19 |
| *Nonforest_dist* | 0.03 | -0.14 | 0.02 | -0.09 | -6.53 | < 0.01 | 1.44 |
| *Floodplains* | 0.12 | 1.12 | 0.09 | 0.18 | 12.86 | < 0.01 | 1.31 |
| *Mean_def* | 0.02 | 0.04 | 0.01 | 0.27 | 5.2 | < 0.01 | 18.1 |
| *SD_def* | 0.01 | -0.02 | 0.01 | -0.21 | -4.28 | < 0.01 | 15.3 |
| *Mean_vs* | 0.01 | -0.26 | 0.08 | -0.06 | -3.4 | < 0.01 | 2.3 |
| *SD_vs* | 0.02 | 1.81 | 0.37 | 0.12 | 4.83 | < 0.01 | 4.2 |
| Simplified model | **R² = 0.523; BIC = 360.5** | | | | | | |
| (Intercept) | - | 1.7 | 0.06 | - | 30.13 | < 0.01 | - |
| *SCC* | 0.17 | 0.69 | 0.05 | 0.20 | 14.59 | < 0.01 | 1.17 |
| *Nonforest_dist* | 0.03 | -0.13 | 0.02 | -0.09 | -5.99 | < 0.01 | 1.39 |
| *Floodplains* | 0.12 | 1.14 | 0.09 | 0.18 | 12.64 | < 0.01 | 1.31 |
| *Mean_def* | 0.06 | 0.02 | 0.002 | 0.13 | 8.56 | < 0.01 | 1.53 |

*SCC* = soil cation concentration; *Nonforest_dist* = distance to the nearest non-forest area; *Floodplains* = floodplains cover fraction; *Mean_def* and *SD_def* = mean and standard deviation monthly water deficit; *Mean_vs* and *SD_vs* = mean and standard deviation monthly wind speed.

**Table S4.** Comparison of Gaussian GLM models to estimate static gap fraction using all samples and addressing spatial correlation. Model goodness-of-fit metrics were calculated out-of-sample from 30 model fits with 10-fold cross-validation. A minimum distance of 100 km was considered when randomly selecting samples for the spatial correlation test.

| Model | All samples (n = 610) | Distance-based sampling to address spatial correlation  (avg. n = 172) |
| --- | --- | --- |
| Regression Parameters | **B estimate** | **B estimate** |
| (Intercept) | 1.65 ± 0.02 | 1.58 ± 0.07 |
| SCC | 0.69 ± 0.01 | 0.65 ± 0.06 |
| Nonforest_dist | -0.13 ± 0.01 | -0.10 ± 0.03 |
| Floodplains | 1.63 ± 0.03 | 1.56 ± 0.19 |
| Mean_def | 0.03 ± 0.001 | 0.03 ± 0.003 |
| Floodplains:Mean_def | -0.11 ± 0.01 | -0.09 ± 0.05 |
| Goodness-of-fit metrics | **Value** | **Value** |
| R² | 0.57 ± 0.009 | 0.56 ± 0.04 |
| RMSE | 1.89 ± 0.32 | 1.71 ± 0.48 |
| RMSE % | 44.9 ± 6 | 41.1 ± 10 |

**Table S5.** Multi-temporal airborne lidar data acquisition information.

| Site | Acquisition Date | Sensor | Flight altitude (m) | Scan frequency (kHz) | Area (ha) |
| --- | --- | --- | --- | --- | --- |
| DUC | 17 Feb 2012 | Optech ALTM 3100 | 850 | 59.8 | 1200 |
|  | 09 Apr 2017 | Optech ALTM 3100 | 850 | 40 |  |
| TAP | 31 Jul 2012 | Optech ALTM 3100 | 850 | 59.8 | 1047 |
|  | 06 Mar 2017 | Optech ALTM 3100 | 850 | 40 |  |
| FN1 | 15 Aug 2013 | Optech Orion M300 | 850 | 67.5 | 992 |
|  | 06 Oct 2018 | Optech ALTM 3100 | 750 | 40 |  |
| BON | 16 Sep 2013 | Optech Orion M300 | 900 | 61.4 | 572 |
|  | 08 Oct 2018 | Optech ALTM 3100 | 750 | 40 |  |
| TAL | 29 May 2014 | Optech Orion M300 | 900 | 61.4 | 480 |
|  | 08 Oct 2018 | Optech ALTM 3100 | 750 | 40 |  |

**References**

1. Brokaw, N. V. L. (1982). The Definition of Treefall Gap and Its Effect on Measures of Forest Dynamics. *Biotropica*, **14**(2), 158. https://doi.org/10.2307/2387750
2. Asner, G. P., Kellner, J. R., Kennedy-Bowdoin, T., Knapp, D. E., Anderson, C., & Martin, R. E. (2013). Forest Canopy Gap Distributions in the Southern Peruvian Amazon. *PLoS ONE*, **8**(4). https://doi.org/10.1371/journal.pone.0060875
3. Espírito-Santo, *et al.* (2014). Size and frequency of natural forest disturbances and the Amazon forest carbon balance. *Nature Communications*, ***5***, 3434. https://doi.org/10.1038/ncomms4434
4. Lobo, E., & Dalling, J. W. (2014). Spatial scale and sampling resolution affect measures of gap disturbance in a lowland tropical forest: implications for understanding forest regeneration and carbon storage. *Proceedings of the Royal Society B: Biological Sciences*, **281**, 20133218. https://doi.org/10.1098/rspb.2013.3218
5. Hunter, M. O., *et al.* (2015). Structural dynamics of tropical moist forest gaps. *PLoS ONE*, **10**(7), 1–19. https://doi.org/10.1371/journal.pone.0132144
6. Silva, C. A., *et al.* (2019). ForestGapR: An r Package for forest gap analysis from canopy height models. *Methods in Ecology and Evolution*, **10***(8)*, 1347–1356. https://doi.org/10.1111/2041-210X.13211
7. Gaulton, R., & Malthus, T. J. (2010). LiDAR mapping of canopy gaps in continuous cover forests: A comparison of canopy height model and point cloud based techniques. *International Journal of Remote Sensing*, **31**(5), 1193–1211. https://doi.org/10.1080/01431160903380565
8. R Core Team. (2018). R: A Language and Environment for Statistical Computing. Vienna, Austria: R Foundation for Statistical Computing. Retrieved from https://www.r-project.org/
9. Bates, D., Mächler, M., Bolker, B., & Walker, S. (2015). Fitting Linear Mixed-Effects Models Using lme4. *Journal of Statistical Software*, **67**(1), 1–48. https://doi.org/10.18637/jss.v067.i01
10. Schwarz, G. (1978). Estimating the dimension of a model. *Annals of Statistics*, *6*(2), 461–464.
11. Abatzoglou, J. T., Dobrowski, S. Z., Parks, S. A., & Hegewisch, K. C. (2018). TerraClimate, a high-resolution global dataset of monthly climate and climatic water balance from 1958-2015. *Scientific Data*, **5**, 1–12. https://doi.org/10.1038/sdata.2017.191
12. Zuquim, G., *et al.* (2019). Making the most of scarce data: Mapping soil gradients in data-poor areas using species occurrence records. *Methods in Ecology and Evolution*, **10**(6), 788–801. https://doi.org/10.1111/2041-210X.13178
13. Hansen, M. C., *et al.* (2013). High-Resolution Global Maps of 21st-Century Forest Cover Change. *Science*, **342**(6160), 850–853. https://doi.org/10.1126/science.1244693
14. Sato, L. Y. (2016). Tecnologia Lidar Para Quantificação Dos Impactos De Incêndios Na Estrutura. PhD Dissertation (Remote Sensing). National Institute for Space Research - INPE. Retrieved from http://urlib.net/8JMKD3MGP3W34P/3M8GSQ2
15. R Core Team (2020). R: A language and environment for statistical computing. R Foundation for Statistical Computing, Vienna, Austria. v.4.0.2. URL https://www.R-project.org/.
